# Koi sleepy disease as a pathophysiological and immunological consequence of a branchial infection of common carp with carp edema virus

**DOI:** 10.1080/21505594.2021.1948286

**Published:** 2021-07-16

**Authors:** Mikolaj Adamek, Felix Teitge, Ilka Baumann, Verena Jung-Schroers, Sahar Abd El Rahman, Richard Paley, Veronica Piackova, David Gela, Martin Kocour, Sebastian Rakers, Sven M. Bergmann, Martin Ganter, Dieter Steinhagen

**Affiliations:** a Fish Disease Research Unit, Institute for Parasitology, University of Veterinary Medicine Hannover, Hannover, Germany; b Department of Virology, Faculty of Veterinary Medicine, Mansoura University, Mansoura Egypt; c Cefas Weymouth Laboratory, International Centre of Excellence for Aquatic Animal Health, Weymouth, Dorset, UK; d South Bohemian Research Centre of Aquaculture and Biodiversity of Hydrocenoses, Faculty of Fisheries and Protection of Waters, University of South Bohemia in Ceske Budejovice, Vodnany, Czech Republic; e Working Group Aquatic Cell Technology and Aquaculture, Fraunhofer Research Institution for Marine Biotechnology and Cell Technology, Lübeck, Germany; f Institute of Infectology, Federal Research Institute for Animal Health, Friedrich-Loeffler-Institut, Greifswald-Insel Riems, Germany; g Clinic for Swine, Small Ruminants, Forensic Medicine and Ambulatory Service, University of Veterinary Medicine Hannover, Hannover, Germany

**Keywords:** Carp edema virus, koi sleepy disease, immunosuppression, hyponatremia, hyperammonemia, metabolome, gills, osmoregulation

## Abstract

Gills of fish are involved in respiration, excretion and osmoregulation. Due to numerous interactions between these processes, branchial diseases have serious implications on fish health. Here, “koi sleepy disease” (KSD), caused by carp edema virus (CEV) infection was used to study physiological, immunological and metabolic consequences of a gill disease in fish. A metabolome analysis shows that the moderately hypoxic-tolerant carp can compensate the respiratory compromise related to this infection by various adaptations in their metabolism. Instead, the disease is accompanied by a massive disturbance of the osmotic balance with hyponatremia as low as 71.65 mmol L^−1^, and an accumulation of ammonia in circulatory blood causing a hyperammonemia as high as 1123.24 µmol L^−1^. At water conditions with increased ambient salt, the hydro-mineral balance and the ammonia excretion were restored. Importantly, both hyponatremia and hyperammonemia in KSD-affected carp can be linked to an immunosuppression leading to a four-fold drop in the number of white blood cells, and significant downregulation of *cd4, tcr a2* and *igm* expression in gills, which can be evaded by increasing the ion concentration in water. This shows that the complex host-pathogen interactions within the gills can have immunosuppressive consequences, which have not previously been addressed in fish. Furthermore, it makes the CEV infection of carp a powerful model for studying interdependent pathological and immunological effects of a branchial disease in fish.

## Introduction

The infection of common carp (*Cyprinus carpio*) gills with a poxvirus named carp edema virus (CEV) has emerged as a serious challenge to the culture of this fish [1,2]. The infection is associated with hypertrophy and proliferation of branchial epithelial cells, occlusion of the branchial intralamellar spaces and fusion of secondary lamellae. Replication of the virus is mainly found in gill tissue rather than in other tissues of carp [3], leading to pathological changes mostly occurring in this organ [4]. This makes CEV infection a potential model for studying pathological effects of a branchial disease in fish.

Geographic separation of carp culture led to the existence of at least two distinctive genogroups (I and II) of the virus; common carps in Europe and North America are infected with genogroup I, whereas genogroup II is more likely to be found in koi from Asia, which are distributed globally due to the ornamental fish trade. In the course of the infection with both genogroups, fish can develop a disease with very characteristic behavioral changes, showing gradually increasing lethargy, which leads to them lying on one side of the body at the bottom of the tank or pond. As a result of this behavior, the disease is called “koi sleepy disease” (KSD).

In KSD-affected carp, pathological changes can be observed in gill tissue, which could suggest respiratory impairment. However, common carp suffering from oxygen shortage are often orient toward the water surface, gasping for air rather than lying at the bottom of the tank. Atlantic salmon, *Salmo salar*, and rainbow trout, *Oncorhynchus mykiss*, suffering from amoebic gill disease (AGD) caused by the amoeba *Neoparamoeba perurans*, experience pathological changes in the gills comparable to those seen in KSD-affected carp. These include epithelial cell hyperplasia of gill filaments, infiltration of inflammatory eosinophils and a subsequent reduction in functional gill area [5]. Initially, gill diseases such as AGD and KSD were thought to cause respiratory failure, supported by the observation of lethargy and respiratory distress in affected fish [6]. For Atlantic salmon and rainbow trout, the hypothesis of respiratory failure as a physiological mechanism responsible for AGD-related mortality has not been confirmed so far [7]. This was considered to be attributed to a significant reserve respiratory capacity in fish, which may respond to hypoxic conditions by modulating the perfusion of gill lamellae [7] via hyper ventilation [8] and by cardiovascular adjustments [9]. AGD-affected salmonids also displayed decreased blood pH (acidosis) and blood hypertension caused by elevated systemic vascular resistance [7], which was related to impaired CO_2_ excretion [7]. The reduced swimming activity observed in AGD-affected salmonids was considered a combined effect of the reduced gill surface area and the cardiovascular compromise noted in affected individuals [7].

However, gill functions are not limited to respiration. Gills are also responsible for ion balance and the removal of metabolic wastes from the amino acid catabolism [10]. Recently, Chang et al. [11] showed that AGD‐associated pathology led to changes in morphology and distribution of chloride cells in the gills of Atlantic salmon. The basal epithelial hyperplasia during progression of an AGD infection coincided with a marked reduction in numbers of Na+/K+‐ATPase positive chloride cells and mRNA expression of the gene encoding for this enzyme [11]. This suggested an impairment of the osmo-regulatory capacity of AGD-affected gills. A severe impairment of the osmoregulation was also considered as a possible cause of death in common carp suffering from koi herpesvirus disease (KHVD) induced by infection with cyprinid herpesvirus 3 (CyHV-3) [12], and could be suggested as a potential cause of mortality during KSD as well. Clinical signs of KSD were shown to be treatable by keeping KSD-affected fish in water containing 0.5% sodium chloride [4].

Surprisingly, in fish, the influence of a gill disease on the release of ammonia has been scarcely studied. This is considered another essential function of gills, which may influence the behavior and survival of the fish. Recently, intoxication with external ammonia has been shown to have a suppressive effect on innate immunity (pathogen recognition, lysozyme, phagocytic and complement activity), and can also influence immunoglobulin composition [14]. These results suggest that an impairment of gill ammonia secretion caused by infection could have an immunological significance, which, up to now, has not been explored.

In order to assess pathophysiological effects of branchial damage caused by the infection of carp with CEV, we analyzed cellular and physiological blood parameters in carp under infection with this virus, and correlated the hematological and physiological changes to pathological findings and virus load in affected fish. In addition, a metabolomic signature of the disease was established. The significance of an impairment of ion regulation for the development of the clinical signs of the disease was examined in an experiment with elevated ion concentrations in tank water. Overall, our data provide a deeper insight into metabolic consequences of the CEV-related branchial disease in carp, and might give some explanation for the sleepy behavior and mortality observed in KSD-affected carp. We further show that the ramifications of gill infection span beyond the metabolic changes and lead to severe immunosuppression.

## Materials and Methods

### Naïve recipient fish

Specific pathogen-free (SPF) common carp from the genetic strains: Amur wild carp (AS), Ropsha carp (Rop), Prerov scaly carp (PS) and koi were used in the infection experiments. Reproduction, growth and keeping conditions were described earlier [3]. Briefly, all carp strains were raised and kept under virus- and parasite-free conditions in an indoor aquaculture recirculation system at 20°C. Fish were fed a commercial feed (Perla Plus, Skretting Norway) at a ratio of 1% body weight per day. Prior to their use in infection experiments, the fish had been confirmed to be free of ectoparasites by means of fresh skin and gill surface smears and examination by light microscopy. They were also confirmed to be free of specific viruses by means of qPCR or RT-qPCR assays as described earlier [3,15]. These assays included the following viruses infecting common carp: CEV, CyHV-3, spring viremia of carp virus (SVCV), and common carp paramyxovirus (CCPV). The fish (n = 176) were used in infection experiments at an age of about 17–24 months and with a body weight between 13 g and 1240 g, depending on the keeping conditions in the recirculation system.

### Infection of naïve carp with carp edema virus in cohabitation experiments

Since CEV cannot be propagated *in vitro* [3], experimental infections with this virus have to rely on the exposure of naïve carp with clinically affected, virus-shedding donor fish obtained from outbreaks of the disease or from persistently infected populations [3]. Before performing the infection experiments, donor fish had been confirmed to be free of ectoparasites as described above. All infection experiments were carried out in accordance with national and international regulations for experimentation with animals and under approval of the Lower Saxony State Office for Consumer Protection and Food Safety (LAVES), Oldenburg, Germany under the reference number: 33.19–425 2-04-16/2144. Based on field observations on fish patients affected by KSD presented to the veterinary diagnostic service at the University of Veterinary Medicine, Hannover, and previous infection experiments, a humane end-point was set up for the infection experiment at a disease stage in which fish were presenting increasing signs of sleepiness to the point in which the most affected individual showed coma-like behavior (CLB), lying on the side of the body with visible respiration but without any motoric response to prodding with a fishing net. In fish patients presented to the above mentioned veterinary diagnostic service, CLB was followed by the death of the KSD-affected individuals. Therefore, all individuals from the infection experiments showing severe sleepiness and CLB were euthanized. During the infection experiment, fish were offered feed up to 1% of their body weight per day.

The cohabitation experiments, CEV VM1, CEV VM2, CEV V, CEV VS and CEV TL were carried out with CEV from the genogroup IIa (Table 1). The cohabitation experiments, CEV VIM1, CEV VIM2 and CEV VII were performed with CEV from the genogroup I. Before cohabitating with a donor fish with a natural infection with CEV, all recipient fish were acclimatized to the water temperature in the infection tanks by lowering the water temperature from 20°C to 18°C by 1°C per day in the case of experiments performed with CEV genogroup IIa, and from 20°C to 16°C by 1°C per day in the case of experiments performed with CEV genogroup I.

**Table 1.** An overview of infection experiments contributing to this communication

Infection abbreviation	Purpose	Carp strains	Virus	Modifications
CEV TL	Virus tissue tropism	Koi	Genogroup IIa	
CEV VM1	Temporal development of the virus infection and of clinical KSD	Koi, AS	Genogroup IIa	
CEV VM2	Temporal development of the virus infection and of clinical KSD	Koi, AS	Genogroup IIa	
CEV V	Assessment of hematological and physiological changes in the serum	Koi, AS	Genogroup IIa	
CEV VB	Assessment of hematological, physiological and metabolic changes in the serum	Koi	Genogroup IIa	
CEV VS	Effect of increased osmolarity in the keeping water on virus infection and KSD	Koi	Genogroup IIa	Salt treatment (rescue)
CEV VIM1	Temporal development of the virus infection and of KSD	AS, PS, Rop	Genogroup I	
CEV VIM2	Temporal development of the virus infection and of KSD	AS, PS, Rop	Genogroup I	
CEV VII	Assessment of hematological and physiological changes in the serum	AS, PS, Rop	Genogroup I	

Cohabitation experiments CEV VM1 and CEV VM2 were performed with carp from the two genetic strains AS and koi to predict kinetics of the clinical signs development (Table 1). Also, the experiment CEV V was also performed with SPF carp from the genetic strains AS and koi (Table 1). These strains were selected because of their large difference in resistance to an infection with CEV genogroup IIa during earlier infection experiments [3]. Subsequently, AS carp were significantly more resistant to the infection and did not develop KSD, while koi were most susceptible and developed severe clinical signs of KSD. The experiments CEV VB, CEV VS and CEV TL were only performed with koi (Table 1). The cohabitation experiments with CEV genogroup I (CEV VIM1, CEV VIM2, CEV VII) were performed on three strains of common carp (PS, AS, Rop).

### Virus tissue tropism and temporal development of the virus infection and KSD

In initial experiments, tissue distribution and temporal development of the virus infection, clinical signs and virus load were measured. In the CEV TL cohabitation experiment, n = 5 koi (13 g – 17 g) were placed in a 400 L tank supplied with a 30 L^–h^ flow-through of tap water three days before cohabitating them with n = 3 donor koi carrying a natural infection with CEV. A tissue library containing brain, gill, kidney, head kidney, liver, spleen, skin, gut, heart and gonads was collected into RNA*later* at day 6 post exposure (dpe) from n = 5 infected fish and kept frozen at −80°C until further analyses. In further experiments, the time course of the development of the virus infection and of clinical signs of KSD was determined. In these cohabitation experiments, CEV VM1 and CEV VM2, n = 10 carp (14 g – 19 g) per strain from the two genetic strains AS and koi were placed into a 400 L tank supplied with a 30 L^–h^ flow through of tap water three days before cohabitating them with n = 3 donor koi carrying a natural infection with CEV. The temperature was maintained at 18°C (±1°C). After 24 h cohabitation, the donor fish were removed from the tank (CEV VM1) and placed into a second tank (CEV VM2) with the same number of naïve carp from the strains koi and AS for an additional 24 h period. In all tanks, the behavior of the fish and the development of clinical signs of KSD were monitored three times per day for a 14-day period. All carp with severe clinical sings of KSD, including severe sleepiness and CLB were removed, euthanized and subsequently their gills, kidney and brains were collected into RNA*later* and kept frozen at −80°C until further analyses. As controls, a mock cohabitation of n = 10 fish per strain was performed with three koi from a separate SPF recirculation system for 24 h.

### Assessment of hematological and physiological changes and immune responses in koi and carp under CEV infection

In the CEV V cohabitation experiment, disease development, hematological and physiological changes in the blood, as well as the expression of genes responsible for immune responses were assessed in gills and the kidney of KSD-resistant AS and KSD-susceptible koi. For this, 24 individuals each from the koi and the AS strain (body weight between 26 g and 43 g) were experimentally infected with CEV genogroup IIa as described above. As non-infected controls, a mock cohabitation of n = 12 fish per strain was performed as described above. At 3, 6, 9 and 13 days post exposure (dpe) by cohabitation n = 6 fish were collected from both genetic strains. The fish were euthanized by immersing them in 0.5 g L^–l^ MS222 and immediately after the fish had stopped moving, heparinized blood was drawn into a S-Monovette (Sarstedt, Germany) from their dorsal aorta. Subsequently, the fish were decapitated and tissue samples from gills, kidney, head kidney, liver and brain were collected into RNA*later* and stored at −80°C for further analysis. From each fish, a gill arch was collected immediately after killing and fixed with 4% phosphate buffered formaldehyde solution and/or Tissue-Tek® O.C.T.™ Compound (Sakura Finetek, Germany) for subsequent histological analysis. At day 6 post exposure, the sampling was biased toward fish showing clinical signs of the disease to better pinpoint the changes related to the severe sleepiness and CLB phenotype.

To confirm the physiological observations and to analyze a possible metabolic impact of KSD on affected carp, another experiment (CEV VB) was performed. For this, a total of six large koi were exposed to koi affected by KSD and sampled 6 dpe. These six koi (360 g – 1240 g), which had been kept separately from other SPF fish for faster growth, were exposed to the same donor fish under the same conditions as described above. Prior to cohabitation, these fish were anesthetized with 0.15 g L^–l^ of MS222 and 0.6 mL of heparinized blood was drawn into a S-Monovette (Sarstedt, Germany) from the dorsal aorta of each fish for pre-infection control analyses. Six days after the cohabitation, all koi were euthanized, blood was collected from the dorsal aorta, and tissue samples were collected into RNA*later* and formaldehyde solution as described above. Additionally, gills from n = 4 fish were collected into DPBS (Sigma, Germany), stored on ice and used for the separation of different populations of gill cells as described below.

### Effect of increased osmolarity in the tank water on virus infection, development of KSD, gill functions, and immune responses

In order to test the hypothesis that a supplementation of sodium chloride (table salt, NaCl) to the tank water could prevent the development of clinical signs of KSD in CEV-infected carp, SPF koi were cohabited with donor koi suffering from KSD for 24 h and then, at 3 dpe, 0.5% NaCl was added to the water in the tank. Such a treatment was recommended to Japanese koi breeders to prevent increased KSD-related mortality^4^. Disease development, hematological and physiological parameters, expression of genes responsible for gill functions and immune responses were assessed in koi from the salt group in comparison to a group of koi exposed to CEV and kept in plain fresh water, and to non-infected control koi kept in salt supplemented and non-supplemented water. In this CEV VS cohabitation experiment, 24 koi individuals (body weight between 28 g and 40 g) were divided into two groups. The first group of 12 fish was cohabitated with fish affected by KSD under the same conditions as described above. After a day of exposure, the koi were randomly divided into two 400 L tanks. In these tanks, 50% of water was changed daily and 3 dpe, in one of the tanks the salinity was increased to 0.5%. This level of salinity was maintained until 6 dpe when the fish were sampled. As non-infected controls, a mock cohabitation of n = 12 fish was performed as described above. One day post exposure, the fish were randomly divided into two 400 L tanks and one tank was supplemented with 0.5% salt as described above at day 3. Six days post exposure by cohabitation, all fish were euthanized by immersing them in 0.5 g L^–l^ of MS222; blood and tissue samples were collected as described above.

### Confirmation of the results using CEV genogroup I

While CEV genogroup IIa affects koi, farmed common carp in Europe are mostly affected with CEV genogroup I. Therefore, the cohabitation experiments, CEV VIM1, CEV VIM2 and CEV VII were performed with CEV genogroup I and three strains of common carp (PS, AS, Rop) in order to confirm the main findings observed during the infection of koi with CEV genogroup IIa. For these, 13 individuals each from the genetic carp strains PS, Rop and the AS (body weight between 31 g and 45 g) were cohabitated as described above at 16°C, with one fish carrying an infection with CEV genogroup I. Later they were allocated to three tanks with n = 5, n = 5 and n = 3 fish per strain, respectively. As non-infected controls, a mock cohabitation of n = 5 fish per strain was performed as described above. At 6 dpe, n = 3 fish were collected from the third tank, the fish were euthanized by immersing them in 0.5 g L^–l^ of MS222, blood was collected from their dorsal aorta and subsequently the fish were decapitated and tissue samples from gills, kidney, head kidney and liver were collected into RNA*later* and stored at −80°C for further analysis. In the other two tanks, the behavior of the fish and the development of clinical signs of KSD were monitored three times per day over a period of 21 days. All carp developing severe sleepiness or CLB were removed, euthanized and subsequently their gills were collected into RNA*later* and kept frozen at −80°C until further analyses.

### Separation of different populations of gill cells

Using Percoll gradient centrifugation, gill cells were separated into three distinct cell populations enriched in pavement cells (PC), goblet cells (GC) or mitochondria rich cells (MRC). For this, gills from n = 4 koi were collected at 6 dpe to DPBS (Sigma, Germany), placed on ice, and subsequently processed using a modified protocol developed by CEFAS, which originally was published by Galvez et al. [16]. Briefly, the gills were digested with 0.25% trypsin (Sigma, Germany) for 15 min.; thereafter, the cells were separated by filtering the digested tissue through a 70 μm cell strainer (Corning, USA). Subsequently, cell populations were separated using a Percoll (GE Healthcare, USA) gradient with three density layer solutions (1.09, 1.06, and 1.03 g mL^-1^), layered in a 15 mL centrifugation tube (Thermo Fisher Scientific, Germany). The cell solutions were layered onto the gradient and separated by centrifuging at 2500 x g for 45 minutes. The resulting fractions were Culture media-1.03 g mL^-1^ Percoll interface = goblet cells (GC), mucus, and cell debris; 1.03–1.06 interface = pavement cells (PC); 1.06–1.09 interface = mitochondria rich cells (MRC). The fractions were collected into separate centrifugation tubes using a pipette and washed by mixing with 5 x volume DPBS, inverting the tube several times and pelleted by a centrifugation at 200 x g for five minutes. Resulting cell pellets were re-suspended in 200 μL of DPBS (Sigma, Germany), subsequently divided into two tubes with 100 μL cell suspension each and 900 μL of Tri-Reagent (Sigma, Germany) or 80 μL ALT buffer (Qiagen, Germany) was added. The samples were kept frozen at −80°C until further DNA or RNA extraction.

### Blood analysis

From the whole blood, hemoglobin content (Hb), hematocrit (Hct), and red blood cell (RBC) and white blood cell (WBC) abundance as well as differential leucocyte counts were performed using standard hematological methods (Houston, 1990). In addition, immediately after collection, 100 µL of blood was collected into a glass capillary and loaded into an OPTI CCA-TS blood gas analyzer (OPTI Medical Systems, USA) equipped with OPTI sensor cassettes E-Ca for measuring blood pH, carbon dioxide partial pressure (pCO_2_), oxygen partial pressure (pO_2_), blood sodium (Na^+^), potassium (K^+^), and calcium (Ca^2+^) levels, as well as the total hemoglobin (tHb) content. In this analyzer, pH is measured via luminescence of an indicator dye, pCO_2_ by means of a CO_2_ sensitive electrode, pO_2_ by luminescence measurements, Na^+^, K^+^ and Ca^2+^ by ion-selective electrodes, and total Hb by means of optical reflection of infra-red light (OPTI Medical Systems). The remaining blood was centrifuged at 600 x g at 4°C for 15 min, the supernatant plasma was collected and immediately frozen at −80°C. Within one week after collection, ammonium levels were measured in the plasma samples by means of a photometric test, (LT Sys, Germany), sodium and potassium levels were determined with a flame photometer (Bayer Diagnostics, Germany), and the protein content was measured with a refractometer (Atago, Germany).

### Virus load and gene expression

Quantitative PCR (qPCR) and reverse transcription – quantitative PCR (RT-qPCR) assays were used for measuring virus load and gene expression. For monitoring the virus load and expression of viral genes as surrogate of viral replication, the gene encoding the P4a capsid core protein of CEV (*p4a*) was used as a target. For host gene expression, three panels of genes were created. The first panel containing cadherin (*cdh1*), occludin (*ocldn a*), Na^+^, K^+^, Cl^−^ transporter (*kir1.1*), Na^+^, K^+^ ATPase (*atp1.1.5*), Rhesus C glycoprotein (*rhcg*), Na^+^HCO3^−^ co-transporter b1 (*ae1b1*), Na+HCO3- co-transporter b2 (*ae1b2*), H^+^ ATPase (*atph+*), carbonic anhydrase (*ca15a*), Na^+^,K^+^,Cl^−^ transporter (*ncc*), sodium hydrogen exchanger 3b (*nhe3b*), aquaporin 3a (*aqp3a*), aquaporin 3b (*aqp3b*), and epithelial Ca2+ channel (*ecac*) was used for monitoring changes in the transcription of the genes responsible for the transport of ions, water, ammonia and gill epithelia integrity in koi sampled during the CEV V experiment. The second panel was used for cell typing after separation of different populations of gill cells and contained: mucin 2-like (*muc2-like), ocldn a*, and *rhcg*. The third panel contained a type I interferon a2 (*ifn a2*), myeloperoxidase (*mpo*), caspase 9 (*casp9*), the surface protein CD4 of T helper cells (*cd4*), surface protein CD8 b1 of cytotoxic T cells (*cd8 b1*), T cell receptor a2 (*tcr a2*) and immunoglobulin M (*igm*), and was used for monitoring general immune responses during CEV V and CEV VS experiments.

### DNA extraction

DNA was extracted from 10 to 25 mg of RNA*later* stored tissues after mechanical lysis in a Tissuelyser II (Qiagen, Germany), using the QIAamp DNA Mini Kit (Qiagen, Germany) in accordance with the manufacturer’s instructions. After isolation, the samples were diluted to 50 ng μL^−1^ and stored at −80°C.

### Quantitative PCR (qPCR)

For detection and quantification of CEV *p4a* DNA, a probe-based qPCR assay developed by the Center for Environment, Fisheries and Aquaculture Science (CEFAS) in Weymouth, UK [17] was performed as described earlier [18]. A brief description can be found in the Supplement, primers and probe sequences are provided in Supplementary Table 1.

### RNA extraction and cDNA synthesis

The total RNA was extracted from 25 mg of RNA*later* stored tissues using Tri-Reagent (Sigma, Germany) in accordance with the manufacturer’s instructions. Any remaining genomic DNA was digested with 2 U of DNase I (Thermo Fisher Scientific, Germany) in accordance with the standard protocol. Synthesis of cDNA was performed from 300 ng of the total RNA using the Maxima™ First Strand cDNA Synthesis Kit (Thermo Fisher Scientific, Germany). A non-reverse transcriptase control was included in the analysis of each sample. cDNA samples were diluted 1:20 with nuclease-free water (Thermo Fisher Scientific, Germany) prior to RT-qPCR analysis.

### Reverse transcription – quantitative PCR (RT-qPCR)

For quantification of viral and host mRNA, a SYBR Green based RT-qPCR was used as described earlier [19]. A short description can be found in the Supplement, sequences or primers are provided in Supplementary Table 1.

## Histology

Histology was used to assess pathomorphological changes of the branchial tissue caused by CEV-infection using the methodology and scoring system described earlier [20]. A brief description can be found in the Supplement.

### In situ *hybridization (ISH)*

*In situ* hybridization of gill tissue was used for visualizing infected cells in branchial epithelium. For ISH, a modified protocol developed earlier by Bergmann et al. [21] was used. A description of this procedure can be found in the Supplement.

### Immunohistochemistry

In order to track changes in tissue expression of tight junction proteins in gill epithelia of carp under infection with CEV, immunohistochemistry staining for tight junction protein-1 (ZO-1) was performed. Gill fragments from n = 6 diseased koi were collected at day 6 post infection (experiment CEV V) into the Tissue-Tek® O.C.T.™ Compound (Sakura Finetek, Germany) and frozen at −80°C. From frozen blocks, sections were cut to a thickness of 3 μm and fixed with ice cold methanol for 10 min. Subsequently, the sections were stained with a rabbit polyclonal ZO1 tight junction protein antibody. For this, the slides were initially overlaid with PBS containing 1% bovine albumin fraction V (Roth, Germany) and 0.2% saponin (Sigma, Germany), and incubated at room temperature for 30 min. Subsequently, the slides were washed with PBS containing 0.2% saponin, and overlaid with the rabbit polyclonal ZO1 antibody (Sigma catalog number AB2272) diluted 1:200 in PBS containing 0.2% saponin and incubated overnight at 4°C. After washing, the slides were overlaid with goat anti-rabbit DyLight 633 antibody (Thermo Fisher Scientific catalog number 35,562) diluted 1:500 in PBS containing 0.2% saponin and incubated for 1 h in the dark at RT. Then, the cells were washed and overlaid with ROTIMount FluorCare DAPI (Roth, Germany) and observed by UV microscopy (Zeiss, Germany). Two types of controls were included: (i) non-infected gills were stained in the same way as samples from the infection experiment; (ii) the primary antibody was replaced by the blocking solution.

### Water content in the brain tissue

The presumed disruption of the osmotic balance in koi suffering from KSD could lead to an increase in the water content in the tissues. Especially vulnerable for this process could be the brain, which is enclosed and limited by the scull. For measuring the amount of water in the brain, a method described by Lisser et al. was used [22]. Brains removed from n = 5 control and n = 5 KSD-affected fish (collected from the fish from experiment CEV VM2) were weighed, dried for 24 h at 70°C and weighed again. The results are presented as a volume of water (mL) per gram of dry weight of the tissue.

### Metabolome analysis

In order to analyze metabolic consequences in koi experiencing the described changes in gill structure under KSD, a metabolome analysis of blood plasma was performed. Plasma samples collected from the larger koi (n = 6) during the CEV VB experiment were analyzed by a global metabolomics approach. Two groups, the samples collected prior to infection and the samples collected 6 days post exposure to fish suffering from KSD were analyzed as biological hexaplicate in a Non-targeted Metabolite Profiling approach by means of gas chromatography-mass spectrometry (GC-MS) and liquid chromatography – hybrid quadrupole time of flight mass spectrometry (LC-QTOF/MS) at Metabolomic Discoveries, Germany.

### Statistical analysis

SigmaPlot 12 software (Systat Software, USA) was used for statistical analysis. Virus load and gene expression data were transformed using a Log10(x) transformation before further statistical analysis to ensure normal distribution and equality of variances. Significant differences (p ≤ 0.05) between infected and non-infected experimental groups were assessed using a t-test, one-way or two-way ANOVA with subsequent pairwise multiple comparisons using the Holm-Sidak method. Graphical data are presented as mean bars (+SD) or box plots of 25%-75% (± minimum and maximum values) with an indication of the median using GraphPad Prism 7 software (GraphPad Software, USA).

## Results:

### Presentation of the disease

#### Target tissues of the virus

**Figure 1.**
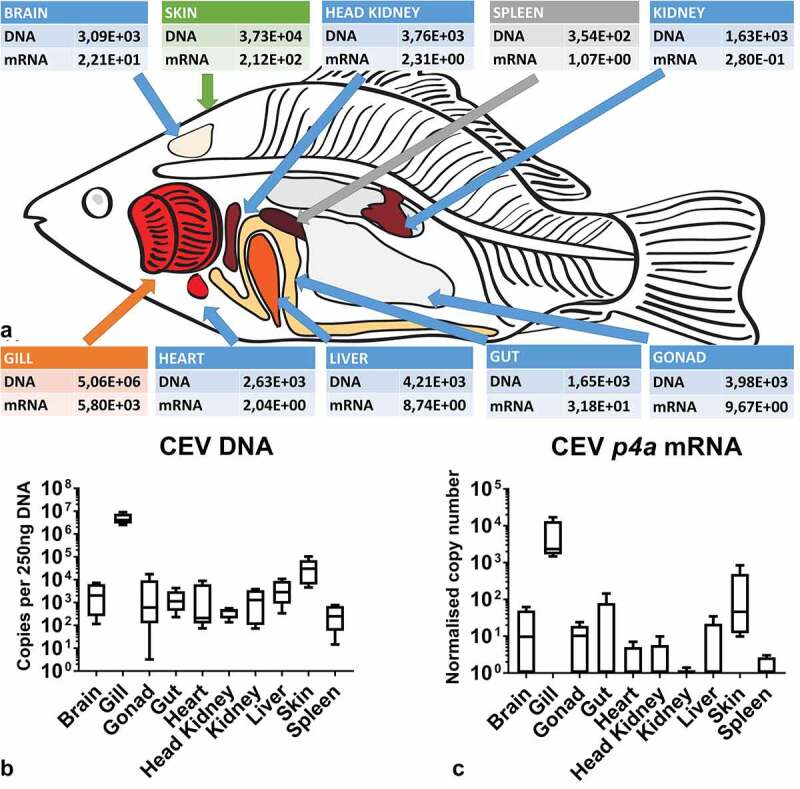
(a) Mean virus load and expression of transcripts encoding the viral P4a capsid core protein in a tissue library of KSD-affected fish showing that gills are the main target organ for the virus. Colors indicate different levels of virus load: orange 10^7^ copies – 10^5^ copies, green 10^5^ copies - 10^4^ copies, blue 10^4^ copies – 10^3^ copies, grey <10^3^. (b) Box plot diagrams presenting 25%–75% (±minimum and maximum values) percentiles and the median as a horizontal line of the virus load in analyzed carp tissues. Depicted is the copy number of viral genome per 250 ng of isolated DNA from n = 5 specimen. (c) Box plot diagrams presenting 25%–75% (±minimum and maximum values) percentiles and the median as a horizontal line of data on the expression of mRNA encoding the viral P4a capsid core protein in normalized copy numbers in analyzed tissues from n = 5 specimen

Screening a tissue library from KSD-affected fish at 6 dpe showed that gills harbored the highest level of virus DNA per 250 ng of isolated DNA (Figure 1a, 1b). The virus DNA level with the mean of 5,060,000 copies was statistically significantly higher than in all other tissues. Gill virus burden was >100 times higher than the virus load in skin, >1,000 times higher than the virus load in brain, gonads, gut, heart, kidney, liver and >10,000 times higher than the virus load in head kidney and spleen (Figure 1). Similarly, gills had the highest expression of *p4a* mRNA encoding the P4a core protein from the CEV virion (5,800 normalized copies). This transcription rate was more than ten times higher than in skin and up to 10,000 times higher in kidney (Figure 1a, 1c).

#### Kinetic of CEV infection and development of clinical signs of KSD

**Figure 2.**
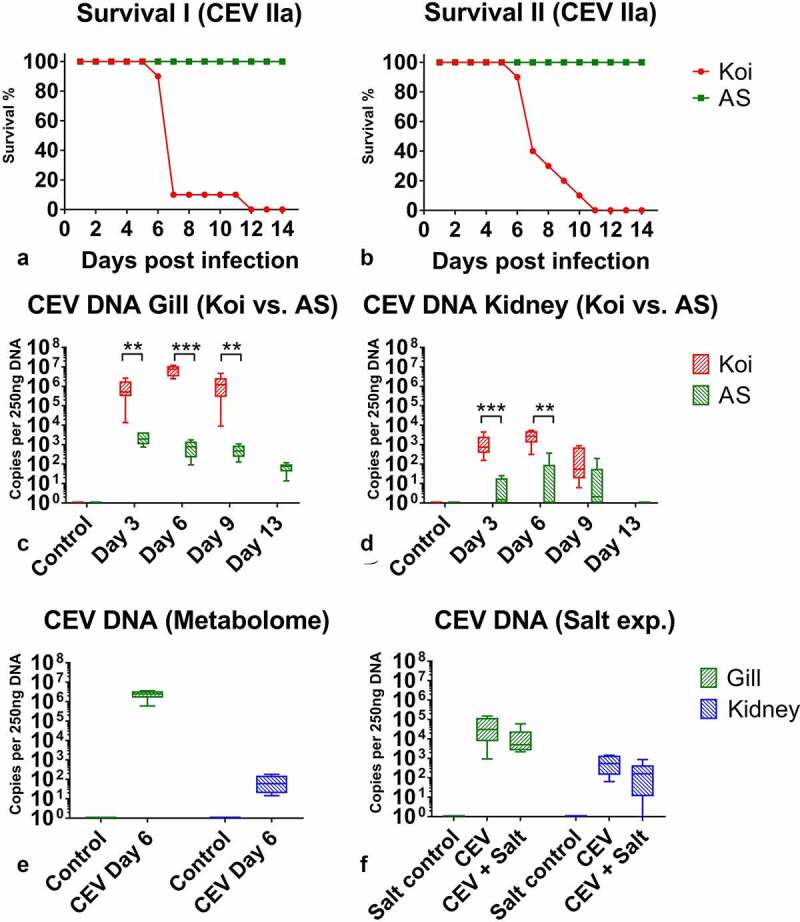
Kinetics of an infection of koi and AS carp with carp edema virus from genogroup IIa during different experiments. (a) and (b) Approximated mortality curves based on the number of animals removed from the experiment when they reached the clinical signs score qualifying for the humane end-point of the experiments. All animals removed presented severe sleepiness or CLB when they were not responding to external stimuli. (c) and (d) CEV loads in gills and kidney of specimen from the koi and AS strains, which show extremely different levels of susceptibility to the infection. Koi were highly susceptible, while AS were highly resistant to the CEV infection and development of KSD leading to mortality. (e) Virus load in the gills and kidney of fish used in the metabolome study. (f) Virus load in the gills and kidney of fish in the salt treatment study. Significant differences between koi and AS are marked with * at p ≤ 0.05, with ** at p ≤ 0.01, with *** at p ≤ 0.001. Mortality data are presented as percentage curves of fish remaining in the experiment. Virus load data are presented as box plots of 25% – 75% percentiles (± minimum and maximum values) with an indication of median as a horizontal line

**Figure 3.**
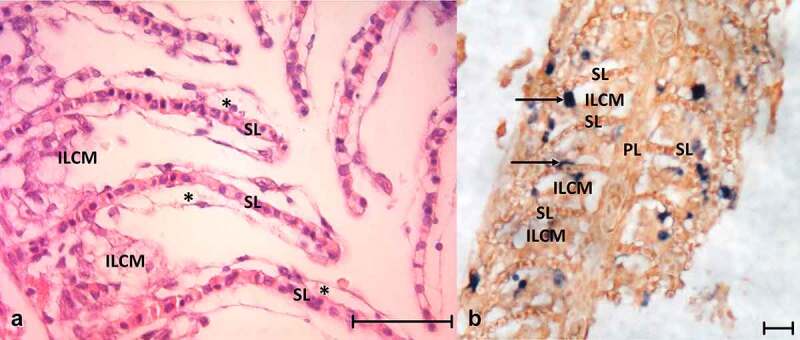
(a) Histology of the gills from CEV-infected fish showing the secondary lamellae edema with lifting of the epithelium (asterisks) and proliferation of cells in the intralamellar spaces. (b) *In situ* hybridization detecting CEV DNA in infected gills; dark blue signal (arrow) indicates CEV-infected cells. ILCM: intralamellar cellular mass, PL: primary lamella of the gill, SL: secondary lamella. Bar 50 µm

After exposure to koi infected with CEV, recipient koi developed severe clinical signs of KSD from 5 or 6 dpe onwards. These clinical signs included severe lethargy, which in some cases rapidly led to coma-like behavior, and the CLB affected specimen were not responsive to external stimuli. Thus, by day 7, most of the koi (90% in tank CEVM1 and 60% in CEVM2) had to be euthanized (Figure 2a, 2b). At 12 dpe, all remaining koi developed severe lethargy or CLB and had to be removed from the tanks, while no visible clinical signs were observed in AS carp throughout the experiments (Figure 2a, 2b). In the control fish not exposed to the virus, no clinical signs were observed and no CEV DNA could be detected in the gills and kidney. However, in AS and the koi, which had been exposed to donor koi infected with CEV in the CEV V experiment, CEV DNA could be detected in gill tissue, with koi harboring higher loads of virus-specific DNA compared to carp from the AS strain (Figure 2c). While in AS carp, approx. 10^2^–10^3^ copies of CEV specific DNA were recorded from 250 ng DNA isolated from gill tissue, the gills of koi harbored 10^6^–10^7^ copies (Figure 2c). In specimens from both strains, the kidney tissue harbored a lower amount of virus, which did not exceed >10^4^ copies. Similar to the gills, in the kidney, the virus load was significantly higher in koi when compared to AS carp (Figure 2 D). All individuals were examined for the development of a coinfection with flavobacteria by means of a qPCR [20]. However, no statistically significant changes were observed in the abundance of flavobacteria on gill tissue during the course of the CEV infection (data not shown). Severe histopathological changes of gill tissue, including a complete occlusion of the intralamellar space by a hyperplasia of epithelial cells, an accumulation of cellular debris and an infiltration of eosinophilic cells were noted in koi sampled at 6 dpe. These changes were not evident in AS carp throughout the observation period (Figure 3a, Supplementary Table 2).

#### Detection of CEV-infected cell populations in branchial tissue

*In situ* hybridization of gill tissue showed that most of the infected cells belonged to branchial epithelium cells of secondary lamellae or were located in close proximity to the secondary lamellae (Figure 3b). The cells occluding the intralamellar spaces expressed no or infrequent signals resembling virus DNA (Figure 3b).

**Figure 4.**
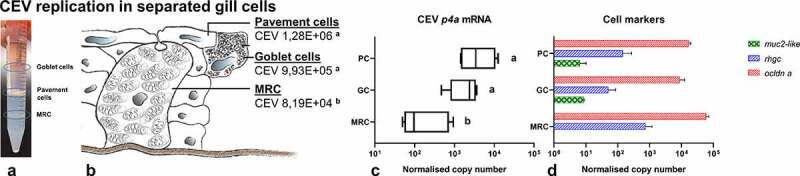
Separation of gill cell populations by density gradient centrifugation, determination of the abundance of cell surface proteins and virus load in separated cell populations. (a) Gill- derived cells separated by Percoll gradient centrifugation. (b) Diagrammatic presentation of distinct populations of pavement cells (PC) goblet cells (GC) and mitochondria rich cells (MRC) in gill tissue. Cells isolated from infected gills harbored different mean levels of virus DNA. (c) Different levels of expression of mRNA encoding the P4a core protein of CEV. (d) Expression of the cellular markers mucin 2-like (*muc2-like*), occludin (*ocldn*), and rhesus glycoprotein type C (*rhgc*) in the different gill-derived cell populations. Letters (a, b) indicate significant differences at p ≤ 0.05 between different cell populations. Expression data for *p4a* are presented as box plots of 25% – 75% percentiles (± minimum and maximum values) with an indication of median as a vertical line. Mean host genes expression is presented as a bar (+SD). The diagram drown by authors based of MRC cells diagram by Degnan et al. ^66^

Using Percoll gradient centrifugation, gill cells were separated into several distinct cell populations (Figure 4a). Three populations were obtained from infected gills, which were enriched with (i) pavement cells, (ii) goblet cells, or (iii) mitochondria rich cells (MRC), respectively (Figure 4b, 4c, 4d). Measurement of the abundance of transcripts of genes encoding the cell marker proteins: mucin 2-like (*muc2-like*), occludin (*ocldn a*) and rhesus glycoprotein type C (*rhgc*) showed that the separated cells were not entirely pure populations (Figure 4d). Importantly, in the MRC-enriched cell population, no expression of the gene encoding the muc2-like protein (goblet cell marker) could be detected, but the highest expression of the MRC marker (*rhgc*) responsible for ammonia excretion was recorded (Figure 4d). This could indicate that this population was the purest and contained ionocytes. By applying this separation method, in the populations enriched with goblet cells and pavement cells, a statistically significantly higher level of viral DNA and *p4a* mRNA was measured than in the population enriched with MRC (Figure 4b, 4c). The level of viral DNA/mRNA was 12 and 7.5 times higher in goblet cells and 16 and 18 times higher in pavement cells than in MRC (Figure 4b, 4c). Still, in all cell fractions, virus-specific DNA and virus-specific mRNA as surrogate for virus replication were detected.

#### Physiological blood parameters in carp under CEV infection

**Figure 5.**
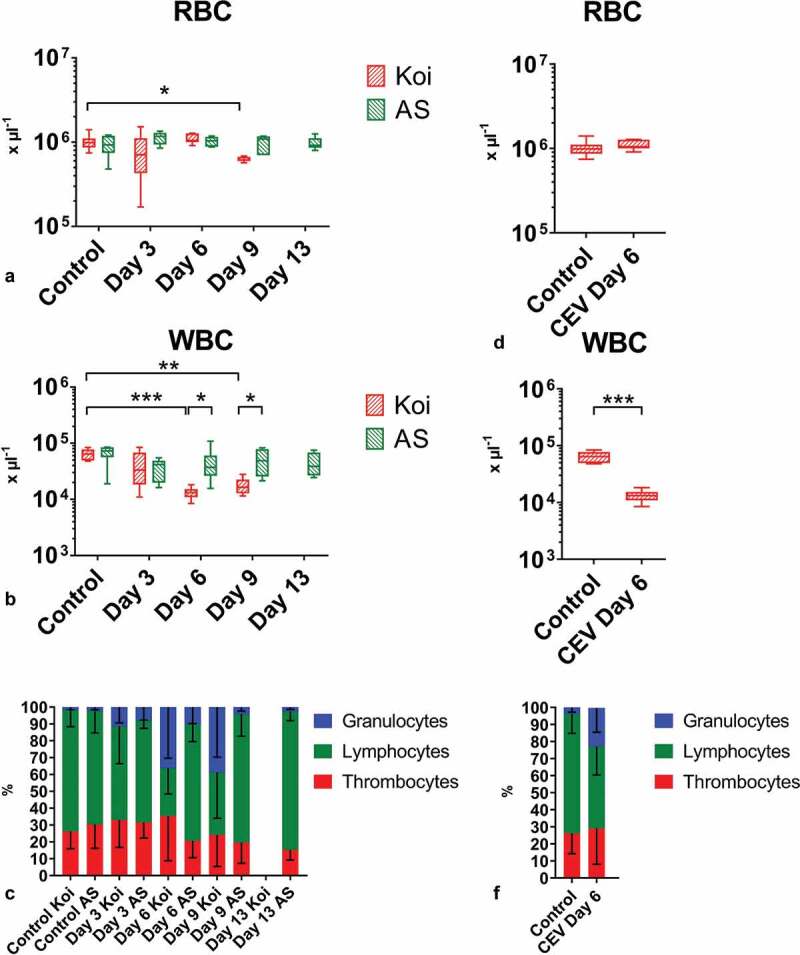
Blood cells in carp from different carp strains under CEV infection. Depicted are total and differential blood cell counts: (a) number of red blood cells (RBC), (b) number of white blood cells (WBC), (c) percentage of granulocytes, lymphocytes and thrombocytes during CEV V experiment in two strains of carp, (d) RBC, (e) WBC, (f) percentage of granulocytes, lymphocytes and thrombocytes in fish used for metabolomics studies. Total and differential blood cell counts show leukopenia and granulocytosis in infected specimen from the KSD susceptible koi strain. Analysis was performed with t-test or two-way ANOVA with strain and time-points as factors. The ANOVA was followed by subsequent pairwise multiple comparisons using the Holm-Sidak method. Significant differences between the control and infected specimens and between strains are marked with * at p ≤ 0.05, with ** at p ≤ 0.01, with *** at p ≤ 0.001. RBC and WBC are presented as box plots of 25% – 75% percentiles (± minimum and maximum values) with an indication of median as a horizontal line. The mean percentage of granulocytes, lymphocytes and thrombocytes are presented as a bar (+SD)

Hematological and clinical chemical parameters were recorded from the blood of AS carp and koi at different time-points post exposure to KSD-affected donor koi and compared to non-exposed AS carp and koi, respectively. While in the blood of AS carp, the level of most of the analyzed hematological parameters was not significantly different from the level of individuals not exposed to KSD-affected koi (Figure 5. Table 2), the blood parameters in koi under CEV infection underwent remarkable changes. In infected koi, the number of erythrocytes and the hematocrit were slightly, but not statistically significantly elevated. The hemoglobin level, however, was significantly increased in samples collected 3 and 6 dpe (Table 2). Most prominent was a clear leukopenia in CEV-infected koi at all sampling dates (Figure 5) and a granulocytosis in the blood of koi sampled at 6 and 9 dpe (Figure 5).

**Table 2.** Blood parameters in carp from the koi and AS strains during a CEV infection. Mild lack of oxygen, severe loss of ions and accumulation of ammonia could be noticed in KSD-affected koi, while not affected AS showed no significant changes in blood parameters. Significant differences between the control and infected specimens are marked in bold and with * at p ≤ 0.05, with ** at p ≤ 0.01, with *** at p ≤ 0.001. Letters (a, b) indicate significant differences between different carp strains at p ≤ 0.05. P – blood plasma, B – whole blood, FP – flame photometer, OPTI – Osmetech OPTI CCA Blood Gas Analyzer, PM – photometer, RF – refractometer, # indicates that sodium level was below levels which can be measured with the equipment

Factor	Instrument			Koi	AS	Koi	AS	Koi	AS	Koi	AS	AS
				Control	Control	CEV 3 dpe	CEV 3 dpe	CEV 6 dpe	CEV 6 dpe	CEV 9 dpe	CEV 9 dpe	CEV 13 dpe
pH	OPTI	B	Mean	7.35	7.35	7.37	7.39	7.34	7.38	7.44**	7.48	7.38
			SD	0.09	0.08	0.06	0.04	0.12	0.03	0.02	0.06	0.09
pCO_2_ (mmHg)	OPTI	B	Mean	22.50	21.67	28.83***a	**24.00b**	24.67	23.50	**24.00a**	**19.83b**	22.50
			SD	2.43	2.94	3.66	2.53	1.75	1.38	2.83	1.83	3.39
pO_2_ (mmHg)	OPTI	B	Mean	41.00	61.50	17.83**	31.17	15.17	35.17	**16.00a**	**48.33b**	41.83
			SD	17.56	21.93	9.17	19.18	6.82	20.07	6.83	20.09	17.05
BE (mmol L^−1^)	OPTI	B	Mean	−12.13	−12.38	−7.88*	−9.22	−11.12	−10.12	−6.98*	−7.53*	−10.80
			SD	3.22	2.25	2.78	2.25	4.91	1.53	2.08	3.05	2.35
tCO_2_ (mmol L^−1^)	OPTI	B	Mean	12.83	12.32	17.20*	14.97	14.48	14.27	16.80*	15.08	13.55
			SD	1.86	0.84	2.24	2.00	5.20	1.29	2.21	2.39	1.07
HCO_3_ (mmol L^−1^)	OPTI	B	Mean	12.12	11.65	16.32	14.25	13.68	13.57	13.05	14.47	12.88
			SD	1.90	0.90	2.20	1.96	5.15	1.23	7.81	2.35	1.11
Na^+^ (mmol L^−1^)	OPTI	B	Mean	139.00	135.67	125.33***a	**137.67b**	<100***a#	**140.33b**	115.75***a	**139.17b**	138.50
			SD	1.67	3.27	8.36	2.50		1.63	11.24	2.99	1.64
Na^+^ (mmol L^−1^)	FP	P	Mean	130.50	132.17	125.20	127.30	71.65***a	**128.63b**	112.40***a	**126.68b**	126.90
			SD	1.37	3.93	4.42	3.65	7.14	4.24	0.57	1.67	3.28
K^+^ (mmol L^−1^)	OPTI	B	Mean	2.73	2.57	**3.50a**	**2.27b**	4.32*a	**2.65b**	1.40	2.57	2.97
			SD	0.36	0.49	1.88	0.55	1.36	0.33	0.36	0.42	0.54
K^+^ (mmol L^−1^)	FP	P	Mean	2.86	3.14	2.11	2.05***	3.86*a	**2.50b**	1.23***a	**2.85b**	3.09
			SD	0.41	0.82	0.23	0.55	0.15	0.26	0.25	0.69	0.61
Ca^++^ (mmol L^−1^)	OPTI	B	Mean	1.34	1.35	1.30	1.32	1.03***a	**1.45b**	**1.27a**	**1.38b**	1.30
			SD	0.13	0.05	0.04	0.08	0.12	0.07	0.11	0.04	0.04
NH_3_ (µmol L^−1^)	PM	P	Mean	225.98	228.55	**276.63a**	**172.12b**	452.66***a	**196.73b**	131.23	154.38	270.30
			SD	100.92	72.57	24.31	20.52	95.66	63.90	20.63	50.44	111.69
Total protein (gL^−^1)	RF	P	Mean	**24.88a**	**28.82b**	25.43	27.18	30.76*	28.42	**22.17a**	**28.74b**	29.36
			SD	1.23	0.94	1.31	4.65	6.09	3.19	0.83	2.37	4.75
tHb (g L^−1^)	OPTI	B	Mean	**87.00a**	**96.83b**	97.20	100.17	86.17**a	**94.67b**	**86.25a**	**95.67b**	98.50
			SD	6.78	5.64	5.72	6.82	29.42	3.50	4.35	11.91	12.90
Hct© (%)	OPTI	B	Mean	26.17a	29.00b	29.20	30.33	31.25**	28.33	26.00	28.83	29.83
			SD	2.14	1.90	1.79	1.97	2.36	1.03	1.41	3.97	3.97

The measurements for blood oxygen indicated lower blood oxygen levels in carp and in particular in koi under CEV infection. Nonetheless, due to a high variation in the measurements, these changes were not statistically significant (Table 2). The blood CO_2_ level was elevated in koi and carp under CEV infection, with significantly higher blood CO_2_-levels in koi sampled 3 and 9 days post exposure to KSD. Blood pH varied only slightly in koi and carp sampled 3 and 6 dpe to KSD, but were elevated in fish sampled 9 dpe. The measurements for total carbonate and hydrogen carbonate levels in blood varied widely, with elevated tCO_2_ and HCO_3_^−^ in samples taken at day 3 and a high variation in the samples collected from CEV-infected koi at 6 and 9 dpe, while in AS carp under CEV infection, the measurements were not significantly different from the values measured in the blood of uninfected individuals (Table 2).

In blood plasma, sodium, potassium and calcium ion concentrations were not different between AS carp under CEV infection and non-infected control individuals. In contrast to this, the sodium concentration levels were severely decreased in koi under CEV infection, with a level of 71.65 ± 7.14 mmol L^−1^ in individuals with clinical signs of KSD sampled at 6 dpe, and 112.40 ± 0.57 mmol L^−1^ at 9 dpe (Table 2). Potassium levels were elevated in several koi sampled 3 and 6 dpe, and the calcium levels decreased in the plasma of koi sampled at 6 and 9 dpe. The total protein content varied widely, but clear differences could not be observed. In addition, the total ammonia concentration in the plasma of AS carp and koi was measured. The ammonia content in the blood of koi suffering from KSD was significantly elevated to 452.66 ± 95.66 µmol L^−1^ at 6 dpe, which was significantly above the level found in the blood of AS carp (196.73 ± 63.90 µmol L^−1^) and non-infected koi (225.98 ± 100.92 µmol L^−1^), suggesting ammonia intoxication (Table 2).

### Expression of genes encoding tight junction and ion transporter proteins in gills

For an analysis of a possible effect of a CEV infection to ion transport across gill cells and paracellular transport, the transcription of genes encoding tight junction and ion transporter proteins were analyzed in gill tissue. A list of genes analyzed and the mRNA expression of these genes measured in koi during a CEV infection are presented in Table 3. A statistically significant downregulation of transcripts was recorded in the gene encoding carbonic anhydrase 15a (*ca15a*) involved in acid-base regulation and Na^+^ uptake, the epithelial Ca^2+^ channel (*ecac*) involved in Ca^++^ transport through the apical membrane of gill epithelial cells. In contrast to this, aquaporin 3a (*aqp3a*), responsible for regulatory volume changes and osmoreception, was upregulated (Table 3). The expression of other genes measured was not changed in a statistically significant manner. These included e-cadherin (*cdh1*) and occludin a (*ocldn a*), responsible for cell contacts, *kir1.1*, a potassium channel orthologous to the mammalian kidney, sodium/potassium/chloride transporters, member 10, tandem duplicate 2 (*nnc*), Na^+^/K^+^ ATPase alpha subunit (*atp1.1.5*) and two sodium bicarbonate cotransporters 1 (*ae1b1* and *ae1b2*) responsible for ion transport, Rhesus c glycoprotein (*rhcg*), sodium hydrogen exchanger 3b (*nhe3b*), V-type proton ATPase catalytic subunit A (*atph+*) responsible for ammonia excretion (Table 3).

**Table 3.** The effect of a CEV infection on mRNA expression of genes involved in gill transport and barrier functions. The onset of KSD is evident with a significantly lower expression of carbonic anhydrase 15a (*ca15a*) and the apical membrane epithelial calcium channel (*ecac*). This indicates a lower sodium and calcium ion import across the plasma membrane of gill cells in KSD-affected carp. Furthermore, CEV infection led to an increase in the transcription of aquaporin 3a (*aqp3a*), which could be related to enhanced water permeability of the gills. Shown are mean ± standard deviation of measurements from n = 6 specimen. Analysis was performed with one-way ANOVA, followed by subsequent pairwise multiple comparisons using the Holm-Sidak method. Significant differences between the control and infected carp are marked in bold and with * at p ≤ 0.05, with ** at p ≤ 0.01, with *** at p ≤ 0.001

**Gene**	**Protein name**	**Function**		**Time point**			
				**Control**	**3 dpe**	**6 dpe**	**9 dpe**
***cdh1***	E-Cadherin	Tight Junction					
			Mean	7,66E+04	1,14E+05	1,08E+05	8,00E+04
			SD	9,40E+03	6,48E+04	5,31E+03	4,28E+04
***ocldn a***	Occludin	Tight Junction					
			Mean	1,04E+04	8,52E+03	3,94E+03	5,91E+03
			SD	2,37E+03	5,71E+03	8,54E+02	2,82E+03
***kir1.1***	Na^+^,K^+^Cl^−^ transporter	Ion transport					
			Mean	1,72E+03	1,13E+03	1,21E+03	1,69E+03
			SD	7,72E+02	6,48E+02	4,46E+02	1,35E+03
***atp1.1.5***	Na^+^,K^+^ ATPase	Ion transport					
			Mean	5,96E+03	6,14E+03	8,96E+03	6,65E+03
			SD	8,04E+02	4,02E+03	9,93E+02	2,20E+03
***rhcg***	Rhesus C glycoprotein	Ammonia transport					
			Mean	7,31E+02	2,99E+03	5,98E+02	2,34E+03
			SD	2,98E+02	3,81E+03	5,77E+02	3,63E+03
***ae1b1***	Na^+^HCO_3_^−^ Co-transporter b1	Ion transport					
			Mean	3,10E+03	2,37E+03	1,69E+03	3,98E+03
			SD	9,49E+02	2,04E+03	8,24E+02	1,55E+03
***ae1b2***	Na^+^, HCO_3_^−^ Co-transporter b2	Ion transport					
			Mean	2,56E+01	1,27E+01	6,06E+01	9,40E+00
			SD	1,01E+01	6,73E+00	9,59E+01	4,28E+00
***atph+***	H^+^ ATPase	Ion transport					
			Mean	7,49E+02	1,20E+03	7,72E+02	2,05E+03
			SD	1,11E+02	9,97E+02	3,40E+02	2,90E+03
***ca15a***	Carbonic anhydrase	Acid – base regulation					
			Mean	7,74E+03	3,68E+03	8,77E+02**	1,62E+03*
			SD	4,87E+03	3,35E+03	**7,02E+02**	**1,53E+03**
***ncc***	Sodium, Postassium, Chloride transporter	Ion transport					
			Mean	8,57E+02	1,81E+03	2,32E+03	2,12E+03
			SD	2,61E+02	1,71E+03	2,41E+03	1,46E+03
***nhe3b***	Sodium Hydrogen exchanger 3b	Ion transport					
			Mean	1,80E+00	2,80E+00	7,80E+00	1,59E+00
			SD	3,32E+00	3,52E+00	1,02E+01	7,63E-01
***aqp3a***	Aquaporin 3a	Water volume regulation					
			Mean	1,00E+04	3,39E+04	5,80E+04*	1,73E+04
			SD	2,57E+03	3,04E+04	**7,21E+04**	6,40E+03
**aqp3b**	Aquaporin 3b	Water volume regulation					
			Mean	1,25E-01	9,92E-02	9,35E-01	7,99E-02
			SD	1,67E-01	7,83E-02	1,84E+00	7,67E-02
***ecac***	Epithelial Ca^2+^ channel	Ion transport					
			Mean	3,38E+02	4,14E+02	7,10E+01**	4,50E+01***
			SD	1,51E+02	3,16E+02	**3,15E+01**	**2,85E+01**

#### Influence of CEV infection on tight junction protein 1

**Figure 6.**
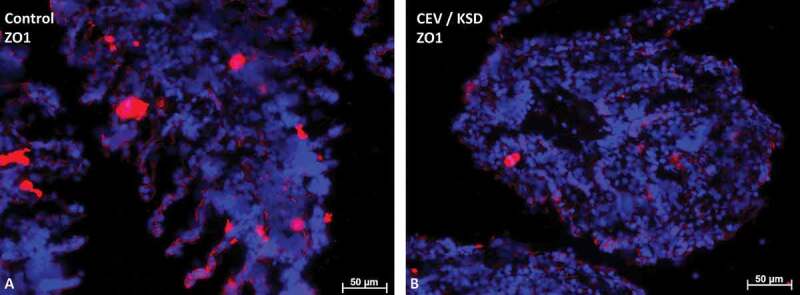
Staining of the tight junction protein-1 (ZO-1) (in red) in gills of CEV not infected (a) and CEV-infected (b) fish, showing severe clinical signs of KSD. Cell nuclei were counterstained with DAPI. CEV-infected gills show a severe occlusion of the intralamellar space, which blocks the contact of epithelial cells (ZO-1 positive) with water. Bar 50 µm

In uninfected carp, a strong labeling of tight junction protein 1 (ZO-1) was present in the branchial epithelium (Figure 6a). In diseased koi suffering from KSD, epithelial cells still expressed this cell contact protein, however, to a lesser extent. In addition, these cells were covered by cells with a weaker expression of ZO-1, which occluded the intralamellar spaces of the secondary lamellae (Figure 6a and 6b).

#### Confirmation of clinical and chemical findings in an experiment with large koi

At 6 dpe, all exposed individuals displayed clinical signs of KSD, disturbed righting behavior, lethargy and frequent lying on one side of the body. The histological examination of the gill tissue revealed a complete fusion of the secondary lamellae, and the intralamellar spaces were occluded by an accumulation of cellular debris and hypertrophy of epithelial cells (Supplementary Table 2). In gill tissue, approx. 10^6^ copies of CEV specific DNA per 250 ng DNA were determined (Figure 2e), while head kidney, liver and kidney of these koi harbored approx. 10^2^ copies of CEV-specific DNA per 250 ng DNA (not shown). In blood samples, the number of red blood cells was not changed (Figure 5d), while the number of white blood cells was significantly (four-fold) reduced, indicating severe leukopenia (Figure 5e). The distribution of white blood cells was also changed. The proportion of granulocytes was increased to the disadvantage of the lymphocytes (figure 5f).

The oxygen tension in circulating blood of koi affected by KSD was significantly decreased and the pCO_2_ was significantly elevated compared to measurements taken from the same individuals prior to CEV exposure. Measurements of blood total carbonate and hydrogen carbonate levels revealed significantly elevated values for these parameters and of base excess calculations in KSD-affected koi compared to measurements prior to CEV infection. The blood pH was elevated compared to the measurements taken prior to infection (Table 4).

**Table 4.** Blood parameters in large koi during CEV infection. A mild lack of oxygen, severe loss of ions and an accumulation of ammonia could be noticed in KSD-affected koi. Shown are mean ± standard deviation of measurements from n = 6 specimen. The analysis was performed with the t-test. Significant differences between the control and infected are marked in bold and with * at p ≤ 0.05, with ** at p ≤ 0.01, with *** at p ≤ 0.001. P – blood plasma, B – whole blood, FP – flame photometer, OPTI – Osmetech OPTI CCA Blood Gas Analyzer, PM – photometer, RF – refractometer. # indicates that the sodium level was below levels which can be measured with the equipment

Factor	Instrument			Big Koi	Big Koi
				Control	CEV 6 dpe
pH	OPTI	B	Mean	7.22	7.34***
			SD	0.07	0.04
pCO_2_ (mmHg)	OPTI	B	Mean	21.50	27.50***
			SD	0.93	2.66
pO_2_ (mmHg)	OPTI	B	Mean	34.00	16.67*
			SD	16.47	1.97
BE (mmol L^−1^)	OPTI	B	Mean	−17.55	−9.98***
			SD	2.46	2.27
tCO_2_ (mmol L^−1^)	OPTI	B	Mean	9.25	15.28***
			SD	1.34	2.24
HCO_3_ (mmol L^−1^)	OPTI	B	Mean	8.59	14.45
			SD	1.36	2.17
Na^+^ (mmol L^−1^)	OPTI	B	Mean	137.38	<100**#
			SD	1.92	
Na^+^ (mmol L^−1^)	FP	P	Mean	130.57	82.10**
			SD	2.75	13.14
K^+^ (mmol L^−1^)	OPTI	B	Mean	2.50	4.60***
			SD	0.38	0.80
K^+^ (mmol L^−1^)	FP	P	Mean	2.30	3.81*
			SD	0.25	1.06
Ca^++^ (mmol L^−1^)	OPTI	B	Mean	1.36	0.89***
			SD	0.14	0.16
NH_3_ (µmol L^−1^)	PM	P	Mean	211.98	658.37***
			SD	66.89	114.57
Total protein (g L^−1^)	RF	P	Mean	26.93	32.15*
			SD	3.92	4.05
tHb (g L^−1^)	OPTI	B	Mean	95.13	107.80*
			SD	5.30	7.43
Hct© (%)	OPTI	B	Mean	28.63	32.40*
			SD	1.51	2.51

In blood plasma, a dramatic drop in the Na^+^ concentration from 130.57 to 82.10 mmol L^−1^ (when measured with a flame photometer) could be noted in koi with KSD compared to the level prior to infection. This was paralleled by a significant reduction in the Ca^2+^ concentration (from 1.36 to 0.89 mmol L^−1^) and a significant increase in K^+^ concentration (from 2.30 to 3.81 mmol L^−1^). Furthermore, in KSD-affected koi, the total plasma protein concentration was elevated and a severe increase in the blood ammonia concentration from 211.98 µmol L^−1^ prior to infection to 658.37 µmol L^−1^ was measured in clinically affected koi (Table 4). Also, hematocrit and hemoglobin content were significantly elevated (Table 4).

#### Influence of CEV infection on blood plasma metabolome

**Figure 7.**
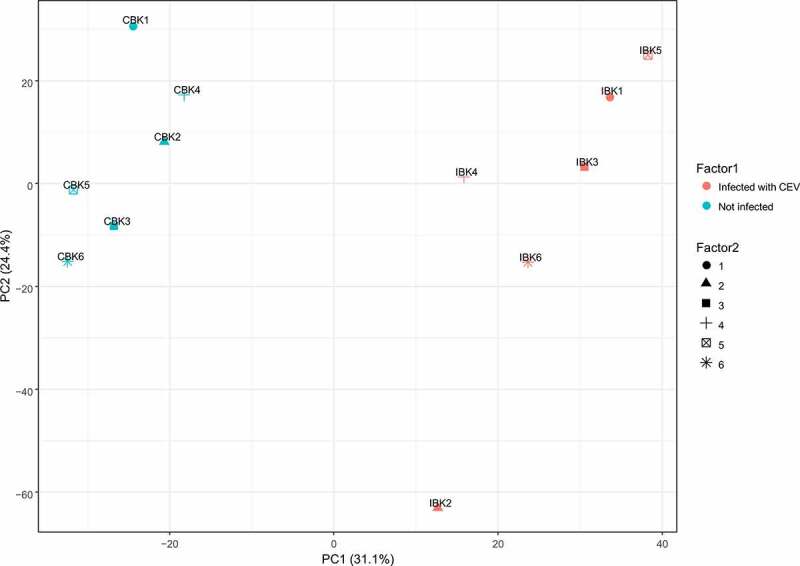
Principle component analyses of blood plasma metabolites from CEV-infected and non- infected koi. IBK1-6 indicates plasma from CEV-infected koi, CBK1-6 plasma from the koi before infection

From all plasma samples from large koi, a total of 2519 metabolites were identified, from which 767 metabolites could be annotated. The metabolism of koi under KSD was clearly different from the metabolism of these individuals prior to infection (Supplementary Figure 1, Supplementary Figure 2). A principal component analysis (PCA) indicated that these metabolic differences were related to the disease (PC1), which separated the plasma samples with the six samples from the infected or non-infected stage clustering each within a group (Figure 7). A list of all metabolites identified is provided in Supplementary file 1 (Snapshot in Supplementary Figure 3), a heat map of all metabolites is provided in the Supplementary file 2 (Snapshot in Supplementary Figure 4). These two files also depict the differences in the abundance of these metabolites between samples from infected versus non-infected koi. In addition, the Supplementary file 3 allows visualization of the metabolome results in the iPath2 an interactive pathways explorer (http://pathways2.embl.de/).

**Figure 8.**
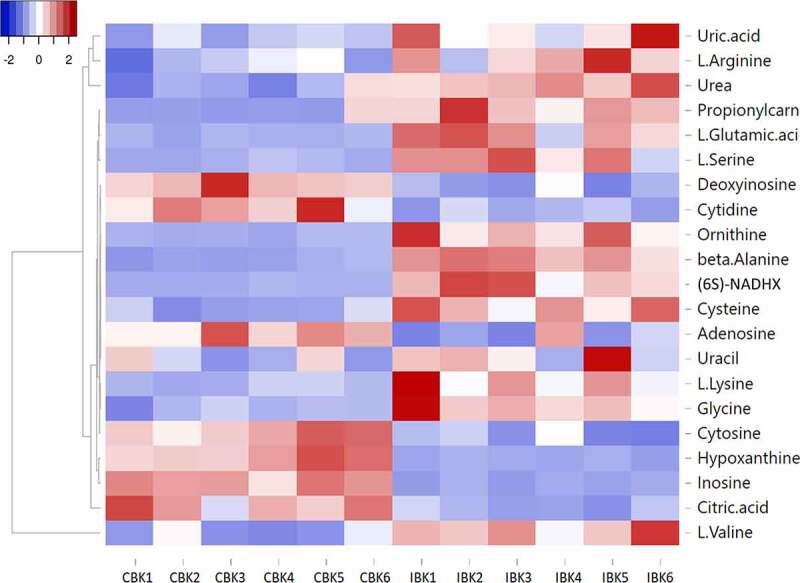
Heat map showing the change in the level of selected metabolites during CEV infection. IBK1-6 indicates plasma from CEV-infected koi, CBK1-6 indicates plasma from koi before infection

Differences in the abundance of metabolites in the plasma of koi affected by KSD versus their abundance in the plasma of these koi prior to infection indicate changes in various biochemical pathways in clinically affected koi. In particular, the pyrimidine and beta alanine metabolism was significantly affected in KSD-affected koi. A reduction in the nucleotide metabolism was indicated by a significant reduction in metabolites of the pyrimidine metabolism like inosine, cytidine or adenosine in the plasma of these koi compared to the metabolism of uninfected koi (Table 5, Figure 8 and Supplementary Figure 5). In addition, beta alanine was increased in the plasma from KSD-affected koi. This metabolite is produced by transamination of one molecule of pyruvate either by conversion of glutamine to α-ketoglutarate by a glutamate-alanine transaminase or by conversion of valine to α-ketoisovalerate via transaminase C (Table 5; Figure 8). The concentration of several amino acids, including serine, lysine, ornithine and valine was increased in the plasma of KSD-affected koi as well, indicating increased levels of free amino acids in the plasma and reduced amino acid catabolism. Among these, in particular, an upregulation of glutamate could be noted (Table 5, Figure 8, and Supplementary Figure 6). In addition, the urea-metabolism was highly upregulated in koi suffering from KSD compared to their metabolism prior to the disease. The concentration of urea was increased, as well as the concentrations of ornithine, a compound from the arginine metabolisms, which is a major pathway involved in urea synthesis in fish (Supplementary Figure 7).

**Table 5.** Effect of a CEV infection on carp metabolism. Ratio and significance of the changes in the levels of selected metabolites in blood plasma of koi during CEV infection

Metabolite	Ratio (CEV infected/control)	P (CEV infected/control)
Inosine	−2,97	9,51016E-05
beta-Alanine	3,08	0,000232878
Hypoxanthine	−3,50	0,000557152
Cytidine	−2,19	0,001732333
Deoxyinosine	−1,61	0,006758606
Cytosine	−1,71	0,00798133
Adenosine	−1,55	0,033103558
Uracil	0,82	0,042072872
Cysteine	1,76	0,000496513
L-Valine	1,36	0,002098849
Ornithine	2,79	0,003292784
Glycine	1,37	0,014477792
L-Glutamic acid	1,87	0,004712621
L-Serine	4,12	0,00410376
L-Lysine	2,03	0,02206657
L-Arginine	0,59	0,013466212
Urea	1,43	0,000238925
Uric acid	1,31	0,04650784
Citric acid	−1,45	0,000751824
Propionylcarnitine	2,26	0,0081727
(6S)-6-beta-hydroxy-1,4,5,6-tetrahydronicotinamide-adenine dinucleotide	4,94	0,00363305

Furthermore, the presence of metabolites from the energy metabolism was altered in the plasma from koi suffering from KSD, with a decrease in citrate and an increase in fructose-1,6 bisphosphate and glucose-6 phosphate, important metabolites from the glycolytic pathway. Furthermore, the carbohydrate metabolites, ascorbate, gluconic acid, glucoronate, xylose, xylitol and glucose, were found more frequently in the plasma of KSD-affected koi compared to the level in healthy koi, indicating an increased mobilization of stored glycogen, which also was indicated by a clear reduction in cellulose, a compound from the starch metabolism (Supplementary Figure 8). Furthermore, the amount of nicotinamide was significantly reduced in the plasma of koi under KSD.

#### Effect of CEV infection on brain water content

**Figure 9.**
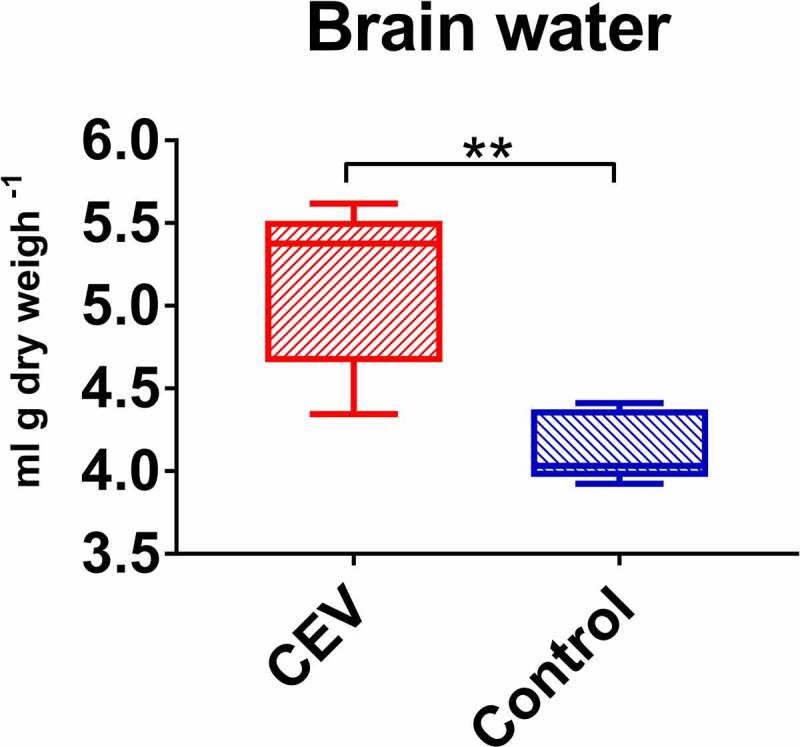
Accumulation of water in the brain of CEV-infected fish. Amount of water in brain per g dry weight is presented as box plots of 25% – 75% percentiles (±minimum and maximum values) with an indication of median as a horizontal line of measurements from n = 5 specimen. The analysis was performed with t-test. Significant differences between the control and infected are marked with * at p ≤ 0.05, with ** at p ≤ 0.0 1, with *** at p ≤ 0.001

The disruption of the osmoregulation in diseased koi influencing the water content of the brain was determined. The CEV infection caused significant accumulation of water by 24% from 4.14 mL g dry weight ^−1^ to 5.14 mL g dry weight ^−1^ (Figure 9).

#### Effect of supplementing tank water with salt on the development of KSD in CEV-infected koi

While koi kept in fresh tap water developed clinical signs of KSD by 6 dpe, the disease could not be observed in koi kept in water supplemented with salt. The histopathological changes were similar in both CEV-infected koi groups and were generally less severe than in other experiments, with moderate hyperplasia of epithelial cells, mild proliferation of intralamellar cells and accumulation apoptotic cells and cellular debris leading to mild occlusion of intralamellar space (Supplementary Table 3). Also, CEV-specific DNA was detected in gills and kidney samples from both groups at a similar level (Figure 2 F), indicating a similar replication of the virus irrespective of the salt supplementation to the tank water. Hemoglobin and hematocrit levels were slightly but not significantly increased in KSD-affected koi kept in fresh water, but not different from control levels in CEV-infected koi kept in water supplemented with salt. Measurements for blood oxygen were slightly reduced in CEV-infected koi kept in fresh water but unchanged in the blood of CEV-infected koi from the salt group compared to non-infected controls. Due to a high variation in measurements, an effect of the CEV infection on blood CO_2_ levels could not be discerned. Blood pH, blood HCO_3_^−^ and total carbonate concentrations were increased in CEV-infected koi kept in fresh water but not in the blood of CEV-infected koi kept in water supplemented with salt (Table 6). Sodium, potassium and calcium ion concentrations were not different between non-infected controls and CEV-infected koi kept in water supplemented with salt, while in CEV-infected koi kept in fresh water, sodium levels were decreased from 130.50 to 80.90 mmol L^−1^, Ca^2+^ levels from 1.34 to 1.06 mmol L^−1^ and the potassium ion concentration was increased from 2.73 to 5.77 mmol L^−1^, respectively (Table 6). Likewise, ammonia levels were not different in the blood of non-infected koi (225.98 µmol L^−1^) and CEV-infected koi kept in water supplemented with salt (246.72 µmol L^−1^), but were significantly increased to 1123.24 µmol L^−1^ in the blood of CEV-infected koi kept in fresh water without added salt. In addition, the serum of non-infected controls and of CEV-infected koi kept in water supplemented with salt had a similar total protein content, while in the blood of CEV-infected koi kept in fresh water, the protein content was significantly increased (Table 6).

**Table 6.** Effect of salt supplementation to tank water on physiological blood parameters of koi during a CEV infection. A mild lack of oxygen, severe loss of ions and an accumulation of ammonia could be noticed in infected koi kept in plain fresh water, while a 0.5% NaCl supplementation to the tank water elevated most of the changes in the blood of CEV-infected koi. Shown are mean and standard deviation of measurements from n = 6 specimen. Analysis was performed with one-way ANOVA, followed by subsequent pairwise multiple comparisons using the Holm-Sidak method. Values in bold and letters (a, b, c) indicate significant differences at p ≤ 0.05 between different treatments. P – blood plasma, B – whole blood, FP – flame photometer, OPTI – Osmetech OPTI CCA Blood Gas Analyzer, PM – photometer, RF – refractometer

Factor	Instrument			Koi	Koi	Koi	Koi
				Control	Control salt 6 dpe	CEV 6 dpe	CEV Salt 6 dpe
pH	OPTI	B	Mean	**7.35b**	**7.12a**	**7.41b**	**7.30b**
			SD	0.09	0.09	0.12	0.08
pCO_2_ (mmHg)	OPTI	B	Mean	22.50	21.33	21.50	22.67
			SD	2.43	2.07	3.15	1.75
pO_2_ (mmHg)	OPTI	B	Mean	**41.00ab**	**46.67ab**	**31.67a**	**58.00b**
			SD	17.56	15.53	9.93	20.59
BE (mmol L^−1^)	OPTI	B	Mean	−12.13	**−20.82a**	**−10.27b**	**−13.52b**
			SD	3.22	3.23	5.71	3.34
tCO_2_ (mmol L^−1^)	OPTI	B	Mean	12.83	**7.48a**	**13.60b**	**11.93b**
			SD	1.86	1.72	3.43	2.29
HCO_3_ (mmol L^−1^)	OPTI	B	Mean	12.12	**6.83a**	**12.98b**	**11.20b**
			SD	1.90	1.70	3.46	2.32
Na^+^ (mmol L^−1^)	OPTI	B	Mean	**139.00b**	**151.17a**	**101.50 c**	**145.83ab**
			SD	1.67	4.96	6.12	4.22
Na^+^ (mmol L^−1^)	FP	P	Mean	**130.50b**	**149.90a**	**80.90 c**	**138.83b**
			SD	1.37	4.29	9.82	3.90
K^+^ (mmol L^−1^)	OPTI	B	Mean	**2.73a**	**3.33a**	**5.77b**	**2.83a**
			SD	0.36	0.78	1.28	1.55
K^+^ (mmol L^−1^)	FP	P	Mean	2.86	3.15	4.07	2.35
			SD	0.41	1.04	1.18	1.75
Ca^++^ (mmol L^−1^)	OPTI	B	Mean	**1.34b**	**1.52a**	**1.06 c**	**1.27b**
			SD	0.13	0.09	0.18	0.16
NH_3_ (µmol L^−1^)	PM	P	Mean	**225.98a**	**256.26a**	**1123.24b**	**246.72a**
			SD	100.92	146.84	304.30	131.01
Total protein (g L^−1^)	RF	P	Mean	**24.88a**	**28.16a**	**42.30b**	**28.17a**
			SD	1.23	3.34	4.42	2.46
tHb (g/L)	OPTI	B	Mean	**87.00ab**	**92.50ab**	**98.50a**	**84.83b**
			SD	6.78	5.74	12.00	5.08
Hct© (%)	OPTI	B	Mean	26.17a	28.00	29.67	25.33
			SD	2.14	1.63	3.72	1.37

#### Immune responses of carp against the infection with CEV

**Figure 10.**
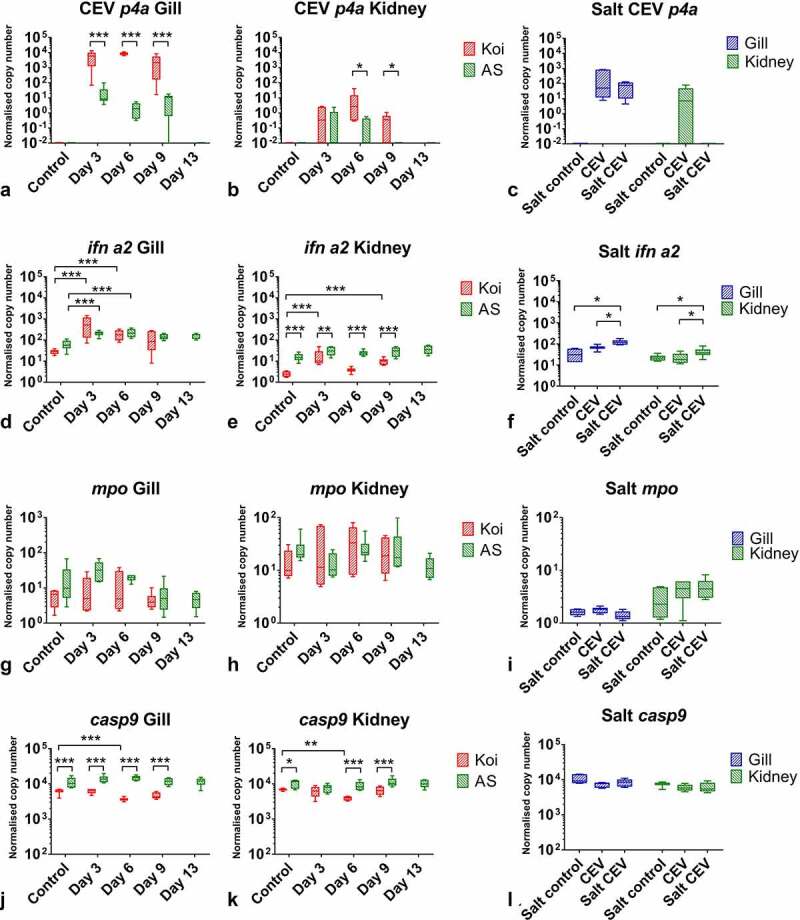
The mRNA expression levels of P4a capsid core protein (*p4a*) of CEV and genes involved in immune responses in carp under CEV infection. (A, B, C) depicted are transcription rates of viral *p4a*, (D, E, F) type I interferon a2 (*ifn a2*), (G, H, I) myeloperoxidase (*mpo*) and (J, K, L) caspase 9 (*casp9*) encoding genes in gills and kidney of koi and AS carp after exposure to koi infected with CEV genogroup IIa and kept in freshwater or water supplemented with 0.5% NaCl. Analysis was performed with one-way or two-way ANOVA with strain and time-points as factors. The ANOVA was followed by subsequent pairwise multiple comparisons using the Holm-Sidak method. Significant differences between the control and infected are marked with * at p ≤ 0.05, with ** at p ≤ 0.01, with *** at p ≤ 0.001. The data are presented as box plots of 25% – 75% percentiles (±minimum and maximum values) with an indication of median as a horizontal line from n = 6 specimen

The expression of the virus-specific *p4a* gene was significantly higher at any time points in gills of koi when compared with AS (Figure 10A). The difference was >100-fold at day 3 and day 9 and >1,000-fold at day 6 post exposure when among koi, most specimens with clinical signs were recorded. Statistical differences between koi and AS were recorded for the *p4a* expression also in the kidney; however, the level of gene expression and the magnitude of differences were much lower (Figure 10B). In the salt rescue experiment, the fish treated and not treated with 0.5% NaCl had a similar *p4a* expression (Figure 10C).

In carp under CEV infection, the type I IFN *ifn a2* expression was significantly upregulated in the gills (Figure 10D) and kidney (Figure 10E). Generally, there was no difference in the response level between koi and AS. Only at 6 dpe was a statistically higher response recorded in kidneys of AS when compared to koi. Also, during the salt experiment, some upregulation of type I IFN expression was recorded (Figure 10F). The expression of the gene encoding for myeloperoxidase was not statistically regulated in the gills (Figure 10G) and kidney (Figure 10H) of koi and AS under CEV infection kept in fresh water as well as during the salt treatment experiment (Figure 10I). In carp under CEV infection, a significant downregulation was observed in the expression of several genes encoding for important proteins for immune response. *Casp9* was downregulated two-fold in the gills (Figure 10J) and kidney (Figure 10K) of CEV-infected koi. This downregulation was also present but not statistically significant in koi in the salt experiment (Figure 10L). Furthermore, CEV-infected AS carp had a statistically significantly higher expression of *casp9* in the gills and kidney compared to infected koi at most of the time points (Figure 10J, 10k)

**Figure 11.**
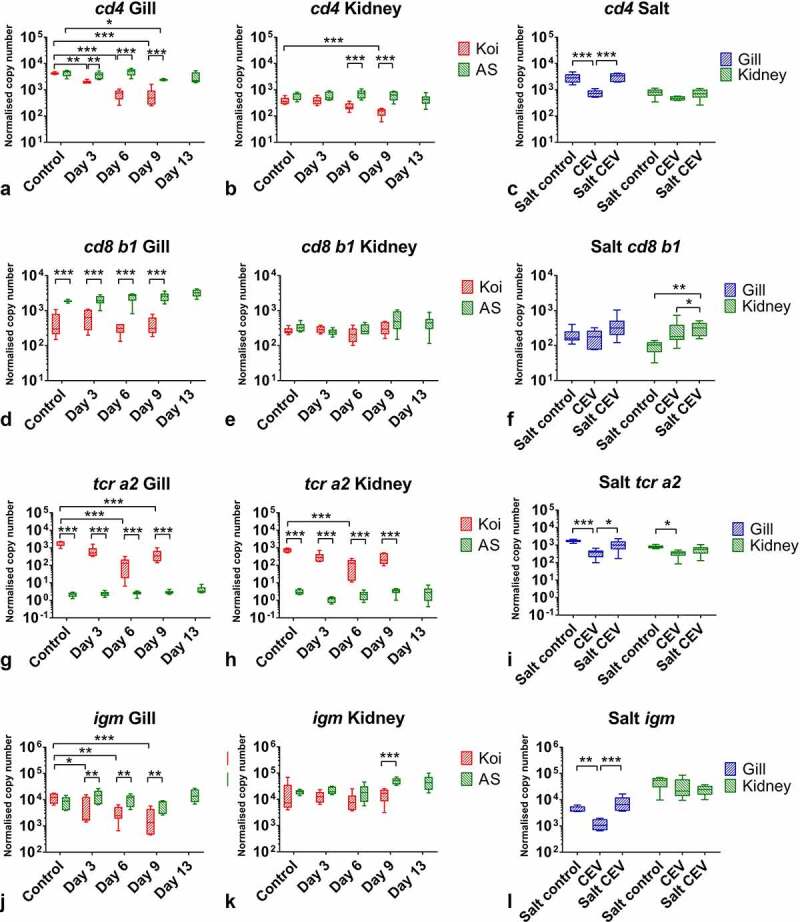
The mRNA expression levels of genes involved in immune responses in tissues of carp under CEV infection. (A, B, C) depicted are mRNA copy numbers of genes encoding surface protein CD4 of T helper cells (*cd4*), (D, E, F) surface protein CD8 b1 of cytotoxic T cells (*cd8 b1*), (G, H, I) T cell receptor a2 (*tcr a2*), (J, K, L) immunoglobulin M (*igm*), involved in adaptive immune responses in gills and kidney of koi and AS carp after exposure to koi infected with CEV genogroup IIa and kept in freshwater or water supplemented with 0.5% NaCl. Analysis was performed with one-way or two-way ANOVA with strain and time -points as factors. The ANOVA was followed by subsequent pairwise multiple comparisons using the Holm-Sidak method. Significant differences between the control and infected carp are marked with *at p ≤ 0.05, with ** at p ≤ 0.01, with *** at p ≤ 0.001. The data are presented as box plots of 25% – 75% percentiles (±minimum and maximum values) with an indication of median as a horizontal line from measurements of n = 6 specimen

Also, the gene encoding for the CD4 receptor of T-cells was downregulated in koi under CEV infection when compared with non-infected controls as well as to the expression in CEV-infected AS (Figure 11A, 11b). The highest downregulation, six- to seven-fold, was measured in gills of infected koi at day 6 and 9 post exposure (Figure 11A). A significant downregulation of the *cd4*-expression was also noticed in the kidney and in gills of CEV-infected fish kept in non-salt supplemented water during the salt rescue experiment (Figure 11C). The salt treated CEV-infected fish had slightly elevated *cd4* expression when compared to the salt treated controls.

The expression of *cd8b1* was also statistically significantly lower in gills of CEV-infected koi when compared with infected AS at any time-points, including the control (Figure 11D, 11e). During the salt treatment, an elevated expression of this gene upon infection was noticed in the kidney of CEV-infected fish and subsequently treated with salt when compared with controls treated with salt as well as fish infected with CEV and without any salt supplementation of the water. (Figure 11F).

Similar to *casp9* and *cd4*, the expression of *tcr a2* was downregulated in the gills of CEV-infected koi 14-fold at 6 dpe and four-fold at 9 dpe (Figure 11G). In summary, this gene was also downregulated in the kidneys of CEV-infected koi at 6 dpe (Figure 11H). A five-fold downregulation of this gene was recorded in the gills during the salt treatment experiment in non-treated fish infected with CEV. In contrast, salt treated CEV-infected fish had an expression of this gene on a similar level as the controls (Figure 11I). The levels of *tcr a2* expression were extremely low in AS, which could suggest an incompatibility of primers for reliably measuring the expression of this gene in this strain of common carp (Figure 11G, 11h). The expression of *igm* followed a similar pattern to the *cd4* and *tcr a2* transcription (Figure 11J, 11k). It was downregulated in the gills of koi under CEV infection, two-fold at 3 dpe and five-fold at 9 dpe (Figure 11J). Also, in this case, salt treatment of the tank water of CEV-infected koi prevented a downregulation of the gene transcription in the gills (Figure 11L).

#### Confirmation of the results by infection of carp strains with CEV from genogroup I

Carp from the PS strain were found to be the most susceptible to CEV from the genogroup I and developed similar clinical signs as observed in koi infected with CEV genogroup IIa. This included the development of severe lethargy and CLB. Therefore, all PS carp had to be removed from the experiment at 12 dpe in the first experiment and 60% of PS fish in the second experiment. In AS and Rop strains, however, only single fish were affected (Supplementary Figure 9a, Supplementary Figure 9b).

When three individuals per strain were used for measuring the virus load (Supplementary Figure 9c) and blood parameters (Supplementary Table 4) at 6 dpe, significant differences were observed. The virus load indicated that two of three PS carp specimens developed a severe CEV infection. These individuals also presented lethargic behavior, while none of the AS and Rop fish showed clinical signs nor had a high virus load (Supplementary Figure 9c). The mean virus load was statistically significantly higher in PS than in AS (340-fold) or Rop (456-fold) carp. The blood parameters were measured with Osmetech OPTI CCA Blood Gas Analyzer and the strongest effect was recorded in the concentrations of sodium, which dropped from 137.67 mmol L^−1^ to 117.33 mmol L^−1^ (Supplementary Table 4). Again, the specimen with the highest virus load had the lowest sodium plasma levels with 108 mmol L^−1^ and 111 mmol L^−1^, while one PS individual as well as all AS and Rop individuals had sodium levels above 129 mmol L^−1^. The virus load in the gills and levels of sodium in blood plasma seemed to be very highly negatively correlated r = – 0.8 to r = – 0.9 (Supplementary Figure 10). This was observed for both CEV genogroup I and CEV genogroup IIa (Supplementary Figure 10).

## Discussion

Establishing the physiological and metabolomic signature of a disease can aid in understanding its pathology, and may lead to the development of novel diagnostic, treatment and prevention strategies. The infection of carp with carp edema virus was selected as a model for investigations into viral gill diseases because this virus predominantly affects this multifunctional organ.

Histopathological changes associated with a CEV infection of carp were mainly restricted to the gills and included proliferative changes of the gill epithelium, and an occlusion of the branchial intralamellar space. Gills are primarily considered as the respiratory organ of fish, and disease-related changes as observed in carp under CEV infection, might directly influence the oxygen supply of affected specimens resulting in functional hypoxia [23]. Fish have evolved various mechanisms to respond to hypoxic conditions. This includes behavioral responses, such as hyperventilation and bradycardia, to keep arterial oxygen saturation at levels seen in normoxia, or elevated hemoglobin concentrations [24]. Likewise, in KSD-affected koi, the Hb concentration was significantly increased. Measurements of blood oxygen levels from koi or carp under CEV infection, however, indicate that these mechanisms could not fully compensate the functional hypoxia in KSD-affected fish. Metabolic responses of fish to such hypoxic situations include a depression in the metabolic rate and a shift in ATP production, with a limitation of aerobic ATP generation and an activation of oxygen-independent ATP generating pathways [25,26]. In zebrafish larvae and adult zebrafish kept under hypoxic conditions, such a metabolic switch was indicated by a down regulation of genes encoding tricarboxylic acid (TCA) enzymes, including citrate synthase, whereas a number of glycolysis pathway genes were upregulated [27,28]. The analysis of metabolites in the present study also revealed a reduction in the citrate level and an increase in fructose-1,6 bisphosphate and glucose-6 phosphate as important metabolites from the glycolytic pathway in the blood serum of KSD-affected koi compared to unaffected koi. This could indicate a metabolic switch toward oxygen-independent pathways of ATP production in KSD-affected koi as well. Carp as a moderately hypoxia-tolerant fish has a higher level of tissue glycogen as endogenous fermentable fuel compared to hypoxia-sensitive rainbow trout [29,30]. The increased level of the carbohydrate metabolites ascorbate, gluconic acid, glucoronate, xylose, xylitol and glucose in the blood serum of KSD-affected koi might therefore indicate an increased mobilization of stored glycogen in response to functional hypoxia induced by the CEV-related branchial pathology. This is further supported by the presence of elevated levels of glucose and glucose-6 phosphate in the serum of KSD-affected koi. Glucose-6 phosphate can be dephosphorylated to glucose to fuel glycolysis [31].

Since glycogen and high-energy phosphates as sources for anaerobic ATP generation may be quickly exhausted [31,32], and an accumulation of metabolic waste from anaerobic metabolisms can have harmful effects on tissues [32], a suppression of the metabolic rate is another strategy to reduce cellular energy consumption in fish under oxygen deprivation [33]. In the KSD-affected carp, the reduction in the pyrimidine metabolism could indicate reduced protein synthesis and growth as major energy-consuming processes [32] in order to meet oxygen shortage related to CEV related gill pathology.

In the present study, increased ammonia levels were observed in carp under KSD, like it is initially noted in the serum of carp under hypoxia or starvation as well [34]. Ammonia excretion in freshwater fish occurs at a proportion of 80–90% across gills epithelia, while kidney, skin or gut account for the remainder [35–37]. The changes to gill anatomy in carp affected by KSD might result in an impairment of ammonia excretion and subsequent elevated body ammonia levels. In various fish species, including several cyprinid fishes, gill morphology is plastic [38–40], and gill surface area may change in response to a developmental program or to environmental factors. In goldfish, a strong correlation of branchial ammonia excretion with the coverage of the intralamellar space by an intralamellar cellular mass (ILCM) [41] was demonstrated. The ILCM hindered branchial ammonia excretion even though in cold adapted goldfish with increased ILCM extrabranchial excretion routes for ammonia via the skin, kidney and gut were employed to a greater extent [41]. In KSD-affected koi with a substantial loss of branchial surface by proliferating intralamellar cells and cellular debris, plasma ammonia concentration was significantly elevated above the level in the plasma of non-affected individuals. This suggests a severe impairment of ammonia excretion in these fish, most likely related to the loss of branchial surface area.

Elevated body levels of ammonia can develop multifactorial toxic effects by interfering with various metabolic processes, including ionic balance [42]. Ammonia acts on the central nervous system, disrupts neurotransmitter metabolism and causes astrocyte swelling [43], which raises intracranial pressure, induces brain herniation, coma and finally death [44]. KSD-affected fish with increased blood ammonia levels experienced a substantial increase in brain water by 24%. The clinical signs in koi affected by KSD, in particular, the disturbed righting behavior and the lying on one body side, might reflect a comatose state, which could be related to brain edema resulting from increased ammonia levels in the brain of the KSD-affected fish.

Along with hyperammonaemia, elevated levels of free amino acids, including increased alanine and glutamine concentrations were observed in the plasma of KSD-affected carp. Similar conditions were reported from the amphibious teleosts *Periophthalmondon schlosseri* or *Boleophthalmus boddaerti*. These fish are at risk of elevated body ammonia levels during their activity on land when branchial ammonia excretion is impaired [45,46]. Increased levels of total free amino acids, in particular, elevated alanine levels developed in tissues and plasma of *P. schlosseri* [47] during amphibious conditions. This indicated that *P. schlosseri* was capable of using amino acids as metabolic fuel, but avoided ammonia toxicity through a partial amino acid catabolism only [45,47]. The increased alanine concentration found in the plasma of KSD-affected carp could indicate that these carp responded to an impairment of ammonia excretion related to the severe branchial pathology by a partial amino acid catabolism as well. Glutamine, which also was increased in the plasma of KSD-affected carp, can be formed by a transamination reaction by transferring ammonia to glutamate, which aids in ammonia detoxification [47–49]. Glutamine can also be used for urea synthesis (reviewed by Anderson [50]). In koi under KSD in the present study, increased urea levels were measured in blood plasma, suggesting urea formation is employed as a strategy for detoxifying increased endogenous ammonia levels in these fish as well.

The most prominent alteration in the physiology of KSD-affected koi was a markedly decreased plasma Na^+^ concentration. In KSD-affected specimens, the plasma Na^+^-concentration dropped to 71.65 ± 7.14 mmol L^−1^, almost half of the Na^+^ concentration in the plasma of non-infected specimens with 130.50 ± 1.37 mmol L^−1^. Current models of ionic and acid-base regulation in freshwater fish postulate the involvement of several types of ionocytes in the transport of ions across the gill epithelium [16,51]. This contrasts to terrestrial vertebrates, where ionic/ osmotic homeostasis is mainly regulated by the kidney [10,52]. However, ionocytes from fish gills are analogous to renal tubular cells from terrestrial vertebrates in terms of function and transporter expression [51]. In gill tissue, we mainly detected CEV in cell populations from the branchial epithelium and observed severe changes to the gill architecture in specimens suffering from KSD. Nonetheless, among the cell populations, which could be isolated from the branchial epithelium, mitochondria rich ionocytes, which mainly carry ion transporters, were found to harbor virus DNA to a much lower amount compared to pavement cells or goblet cells. Likewise, the transcription rate of genes encoding transporters responsible for Na^+^ uptake and H^+^ excretion (*nhe*), sodium/potassium/chloride co-transport (*ncc*) or sodium bicarbonate co-transport (*ae1b 1* and *2*) was not changed in gill tissue of KSD-affected carp.

Based on these observations, the dramatic hyponatremia in KSD-affected specimens could not be linked to a down-regulation of transporter transcription/activity. Instead, it could be related to increased passive losses of ions from infected gills due to increased paracellular permeability of the infected branchial epithelium. In gills of KSD-affected koi, a lower abundance of occludin transcripts and a lower content of ZO-1 protein were found in comparison to non-infected koi. This suggests some “loosening” of the gill epithelium in KSD-affected koi. Gill occludin abundance was found to be sensitive to various environmental and systemic variables, including low water pH [53], feed deprivation [54], or high circulating cortisol [54], while in particular, low water pH and increased cortisol levels resulted in a “tightening” rather than a “loosening” of the epithelial barrier [55].

The loss of sodium severely affects metabolic processes, including the functioning of neuronal cells. In mammals, hyponatremia may cause fatal brain swelling, coma, cerebral hypoxia and a loss of vegetative functions [56]. In rainbow trout exposed to silver contamination, the loss of internal Na^+^ and Cl^−^ led to an osmotic imbalance between plasma and tissues, which led to hemo-concentration and eventually to death caused by circulatory failure [57]. In KSD-affected koi, increased plasma protein concentrations could indicate hemo-concentration in response to the ion losses in these fish as well. The depletion of plasma sodium, however, was abolished when koi under CEV infection were kept in water supplemented with 0.5% sodium chloride, which significantly reduced the steep osmotic gradient between carp serum and environmental freshwater [12]. In addition, in the CEV-infected koi kept in salt supplemented water, plasma ammonium, bicarbonate, carbonate, pCO_2,_ and pO_2_ were not significantly altered compared to non-infected specimens, and the clinical signs of KSD, including CLB, were not observed. This salt treatment was earlier proposed to fish farmers for treating KSD-affected koi and protecting affected specimens from mortality [4]. Likewise, elevated ambient Cl^−^ concentrations protected rainbow trout from silver-induced toxicity [58] by circumventing the inhibition of both Na^+^ and Cl^−^ influx caused by blocking the Na^+^, K^+^-ATPase activity [58]. In the gills of KSD-affected koi, the abundance of transcripts of this and other ion transporters was not altered compared to non-infected koi, but a reduction in occludin transcripts was seen. Therefore, the hypothesis of an increased passive loss of ions over a “loosened” branchial epithelium cannot be abounded in CEV-infected koi; however, the mechanism responsible for this ion loss needs further analysis.

Our results could also suggest that hyponatremia and hyperammonemia in KSD-affected carp could have immunological consequences. An exposure of fish to high concentrations of ammonia in ambient water can change blood morphology including lowering the numbers of red and white blood cells as well as the hemoglobin content of blood [59]. Numbers of WBC and RBC also dropped during CEV infection in clinically affected specimens at 6 and 9 dpe, respectively. Interestingly, the probable immunotoxic effect of hyponatremia and hyperammonemia was not uniform on all populations of WBC. Differential blood cell counts documented a clear increase in the proportion of granulocytes to the disadvantage of lymphocytes. Further RT-qPCR analyses indicated a significant lowering of the abundance of *cd4* and *igm* transcripts in the gills and kidney of KSD-affected koi, but not of *cd8* transcripts, suggesting a change in the presence of different lymphocyte subsets in these tissues. In previous studies, increased ammonia concentration in ambient water resulted in an up-regulation in the expression of inflammatory cytokines, including TNF alpha, IL1b in *Pelteobagrus fulvidraco* [13], and a down-regulation of TLR 2 and TLR3 signaling and apoptosis in *Hemibarbus maculatus* [14], as well as of *igm* and complement *c3* mRNA expression in *Pelteobagrus vachellii* [60]. These findings support the view of an ammonia-related immunosuppression on KSD-affected carp.

In mammals, the downregulation of CD4 is the linchpin of disarming actions against the immunity of several poxviruses. It is unclear whether the down-regulation of *cd4* and *igm* transcription in KSD-affected koi depended on an immunosuppressive effect of hyperammonemia in carp. Thus, clinical signs associated with the infection, enabling the virus to more effectively suppress immune responses of common carp should be further explored. In koi exposed to increased ambient salt, the virus level did not drop significantly compared to koi kept at low salt; however, *cd4* and *igm* expression were not different in these fish compared to non-infected controls. The suppression of T-cell and B-cell responses together with a downregulation of mucin production may be a reason for ubiquitous secondary infections accompanying CEV during outbreaks of KSD [1,2,61]. KSD-affected fish were frequently found co-infected with both bacterial and parasitic pathogens [1,20,62,63]. Our earlier research showed that CEV can, for instance, promote the development of secondary gill infections caused by *Flavobacterium branchiophilum* [20]. Coinfections can exacerbate pathophysiological changes associated with the disease, as was recently shown in smolts of sockeye salmon (*Oncorhynchus nerka*), where the insults to the skin caused by salmon lice and infectious hematopoietic necrosis virus (IHNV) coinfections synergistically caused an osmoregulatory dysfunction with an influx of sodium and chloride from sea water into the fish body [64]. Also, during CEV/*F. branchiophilum*, co-infections increased histopathological changes and mortality was recorded, hinting at synergistic actions of the co-pathogens [20].

## Concluding remarks

**Figure 12.**
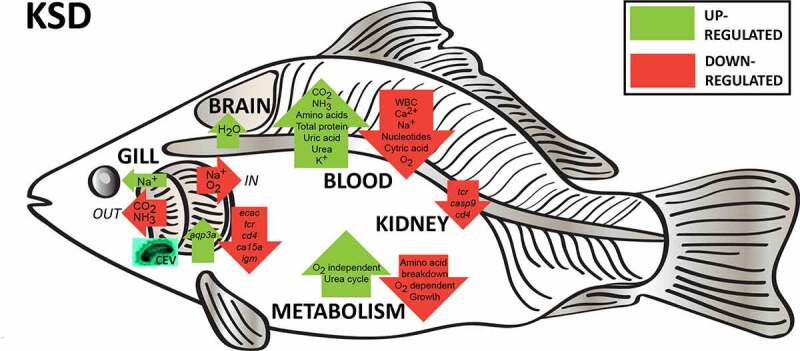
A summary of current results showing that koi sleepy disease is triggered by the gill pathology caused by the infection with carp edema virus. This impairs the respiratory, excretory and osmoregulatory function of gills, leading to a decrease in blood oxygenation and increase in CO_2_ content. This is compensated by a shift from oxygen-dependent to oxygen-independent metabolism and restricted growth of fish, which is reflected in a decrease in citric acid and nucleotide content in blood. Increased loss of ions via damaged gills causes hyponatremia and hypocalcemia. Further cell damage initiates hyperpotassaemia. Impairment of ammonia secretion induces hyperammonemia. This leads to a stop in ammino acid breakdown and increased urea cycle, causing the increase in amino acids, urea and uric acid content in blood. Hyponatremia and hyperammonemia could be associated with the influx of water to the brain and immunosuppression shown as downregulation of *cd4, casp9, tcr a2* expression in gills and kidney and *igm* in gills

We comprehensively analyzed physiological, metabolic and partial immunologic responses of a freshwater fish to virus-induced changes in the gills, the conclusions from the results are summarized in Figure 12. A metabolome analysis of blood plasma underlined that carp as a moderately hypoxia insensitive fish compensate the reduction in oxygen uptake related to a loss of functional gill surface or increased diffusion distance between environmental water and blood plasma by metabolic adaptations, including a reduction in energy demanding processes such as nucleotide metabolism. However, CEV infection was also associated with a severe disturbance of ammonia excretion and the ionic homeostasis in KSD-affected koi. Differently to terrestrial animals, in most fish species, the gills are the main osmoregulatory organ with minor contributions from the intestine and kidney. Clinical signs of KSD could be solved by supplementing the ambient water of CEV-infected carp with sodium chloride. We therefore speculate that branchial diseases affect fish mainly by a disturbance of the complex branchial processes of ionic transport, nitrogen excretion and acid-base balance rather than by an induction of hypoxia. This could support the earlier hypothesis that gills primary evolved in fish for ion exchange and perhaps for ammonia secretion and not for respiration [65]. Importantly, our data allow us to link the disturbance of physiological processes to a suppression of T- and B-cell immune responses observed in specimens suffering from KSD. Thus, the pathophysiological distress related with KSD is causing comatose (sleepy) behavior but also fosters secondary infections, which increase the severity of clinical signs and subsequently lead to the death of the CEV-infected animal. This documents complex host-pathogen interactions, which previously had not been addressed in fish. These observations make CEV infections in carp a powerful model for studying the interdependence of pathological and immunological processes in a branchial disease in fish.

## Supplementary Material

Supplemental Material

## Data Availability

All relevant data are to be found in the manuscript and its Supporting Information files. Any additional raw data are available upon reasonable request.
