# Supplementary material for: Koi sleepy disease as a pathophysiological and immunological consequence of a branchial infection of common carp with carp edema virus
Source: Virulence. 2021 Jul 16;12(1):1855–83. doi: 10.1080/21505594.2021.1948286 (PMC8288041; doi:10.1080/21505594.2021.1948286)
Supplement: Supplemental Material [file KVIR_A_1948286_SM6432.zip › supplementary/KVIR_2021_0052R1_Supplementclean_.docx]

**Supplement**

**Quantitative PCR (qPCR)**

For detection and quantification of CEV *p4a* DNA, a probe-based qPCR assay was performed using the primers CEFAS_qF: AGTTTTGTAKATTGTAGCATTTCC, CEFAS_qR: GATTCCTCAAGGAGTTDCAGTAAA and the double-labeled probe [FAM]-AGAGTTTGTTTCTTGCCATACAAACT-[BHQ1] were used. The reaction mix contained 1× Maxima Probe qPCR Mastermix (Thermo Fisher Scientific, Germany), 500 nM of each primer, 200 nM of the probe, 5 μL of template DNA and nuclease-free water to a final volume of 20 μL The reaction was performed in a Stratagene Mx3005P thermocycler (Agilent, USA). The amplification program included an initial denaturation at 95 °C for 10 min, followed by 40 cycles of denaturation at 95 °C for 30 s and annealing at 60 °C for 30 s. A standard curve from 10^1^ to 10^7^ copies of the *p4a* gene fragment was used for quantification of the copy number of CEV specific DNA in each sample. The results for virus load are presented as the number of virus specific DNA copies per 250 ng of total DNA.

**Reverse transcription – quantitative PCR (RT-qPCR)**

For quantification of viral and host mRNA, a SYBR Green based RT-qPCR was used. The brief description can be found in the Supplement, sequences or primers are provided in Supplementary Table 1. Reactions were performed in duplicate using the Maxima SYBR Green 2× mastermix (Thermo Fisher Scientific, Germany) in a Stratagene Mx3005P thermocycler (Agilent, USA) or StepOnePlus thermocycler (Applied Biosystems, Germany). The reaction mix was prepared as follows: 1× Maxima SYBR Green mastermix (with 10 nM of ROX), 0.2 μM of each primer (sequences in Supplementary Table 1), 5.0 μL of 20× diluted cDNA and nuclease-free water to a final volume of 20 μL. The amplification program included an initial denaturation at 95 °C for 10 min, followed by 40 cycles of denaturation at 95 °C for 30 s, annealing at 55 °C for 30 s and elongation at 72 °C for 30 s. A dissociation curve was performed at the end of each run. For quantification, recombinant DNA plasmid standard curves from 10^1^ to 10^7^ gene copies were prepared and used for quantifying the copy number from each sample.

For normalization of expression, the gene encoding the 40S ribosomal protein S11 (*40S*) and elongation factor 1 alpha (*ef1a*) were used as reference genes. The level of gene expression is shown as the copy number of the gene normalized against 1 × 10^5^ copies of the 40S and 1 × 10^5^ copies of the ef1a (normalized copy number) using the following formula:

Normalized copy number = mRNA copies per PCR for target gene / [(mRNA copies per PCR for reference gene 1 / 10^5^) + (mRNA copies per PCR for reference gene 2 / 10^5^)]

**Histology**

Histology was used to assess pathomorphological changes of the branchial tissue caused by CEV-infection. Gill samples fixed with 4% buffered formaldehyde (Roth, Germany) were stored for 24h at 4 °C. The samples were dehydrated in a series of graded ethanols and embedded into paraffin wax in accordance with a standard laboratory protocol. From paraffin blocks, sections were cut to a thickness of 3 μm and stained with hematoxylin and eosin (HE). To evaluate morphological changes of the branchial tissue, a semi-quantitative scoring system was applied. The following changes were recorded: Thickening, hyperplasia of epithelial cells, lifting of the epithelium, proliferation of the intralamellar cellular mass, presence of cells with edema or foamy contents, number of apoptotic cells and presence of infiltrating granulocytes. The severity of the changes was scored using a scale from 0 to 3: 0 – indicates no changes, 1 – mild changes, 2 – moderate changes, 3 –severe changes.

***In situ* hybridisation (ISH)**

Selected (n = 3) formalin fixed and paraffin embedded tissue sections of gills collected from koi at 6 dpe (from experiment CEV V) were mounted onto Superfrost glass slides (Thermo Fisher Scientific, Germany) and treated with proteinase K (100 μg mL^-1^) in TE buffer for 20 min at 37 °C. Thereafter, they were fixed again by 95% ethanol, followed by 100% ethanol for 1 min. After air drying, sections were covered with a hybridization mix (ISH-M) containing 4 x standard saline citrate (SSC), 50% formamide (v / v), 1 x Denhardt’s reagent, 250 μg yeast tRNA mL^-1^ and 10% dextran sulfate, and incubated for one hour at 42 °C in a humid chamber. DIG-labeled probes were obtained with CEFAS_CEV_F ATGGAGTATCCAAAGTACTTAG, CEFAS_CEV_R CTCTTCACTATTGTGACTTTG primers using a routine protocol described in the “Nonradioactive *In Situ* Hybridization Application Manual” (Roche, Switzerland). The DIG-labeled probe mix (5 μL in 200 μL ISH-M) was applied to each section, placed on the *in situ* plate of a thermal cycler (Eppendorf Mastergradient, Germany), and heated up to 95 °C for 5 min for denaturation of the DNA. The slides were cooled down on ice for 2 min and then incubated overnight at 42 °C in a humid chamber for hybridization. Afterwards, the slides were washed with 2x SSC for 10 min. For removing non-specific bound probes, slides were incubated in 0.4 x SSC at 42 °C for 10 min. Sections were counterstained with Bismarck-Brown Y and observed under a microscope.

**Supplementary Table 1.**

| Target | Primer/probe | Primer/Probe Sequence | Accession number |
| --- | --- | --- | --- |
| *p4a* | CEV_qF1 | TTTAGGAGGACAAGTAAAGTTACCA | KM283182 |
|  | CEV_qR1 | GCAAGTTATTTCGATGCCAAC C |  |
|  | CEV_probe1 | FAM-CCAGCTCCTACAAGGAAAGCAATTGA-BHQ |  |
| *40S* | q40S.FW1 | CCGTGGGTGACATCGTTACA | AB012087 |
|  | q40S.RV1 | TCAGGACATTGAACCTCACTGTCT |  |
| *ef1a* | Cyca_EF1a_qF2 | ACAACCCCAAGGCTCTCAA | AF485331 |
|  | Cyca_EF1a_qR2 | CCGCCAACTTTCTTCTCAAC |  |
| *ifn a2* | IFN_I_2_gsp_qF2 | GATGAAGGTGCCATTTCCAAG | AB376667 |
|  | IFN_I_2_gsp_qR3 | CACTGTCGTTAGGTTCCATTGCTC |  |
| *mpo* | Cyca_MPO_qF1 | GGACTCTCCGCCCCTAAGAA | AB429306 |
|  | Cyca_MPO_qR1 | ACAAAAGGCTCTGCGACACC |  |
| *casp9* | CycaCASP9_qF1 | CGAGAGGGAGTCAGGCTTTC | EC394517 |
|  | CycaCASP9_qR1 | TCAGAAGGGATTGGCAGAGG |  |
| *cd4* | Cyca_CD4_qF1 | CGTGGACATCTGGCTTTGTG | DQ400124 |
|  | Cyca_CD4_qR1 | TTTGGTTTTGCGTCGTCTGT |  |
| *cd8 b1* | Cyca_nCD8b1_qF2 | CGGCTCGGAAACTATCACCT | EU025120 |
|  | Cyca_nCD8b1_qR2 | GAGTGGCGGACAGGTTTTCTC |  |
| *tcra2* | Cyca_tcra2_qF1 | CAGTGCGATATGCAGAACGAAGT | EU025123 |
|  | Cyca_tcra2_qF1 | TTGACTGGATGATCCTTGCTGA |  |
| *igm* | Cyca_igm_qF1 | CACAAGGCGGGAAATGAAGA | AB004105 |
|  | Cyca_igm_qR1 | GGAGGCACTATATCAACAGCA |  |
| *cdh1* | CycaCDH_1_qF4 | TCAGGACAGCTCCATTCAAG | - |
|  | CycaCDH_1_qR4 | AACAGCAGAGCCAAGATTCC |  |
| *ocldn* | Cyca_Ocln_A_qF2 | GGCTATGGAATGGGTGGAG | - |
|  | Cyca_Ocln_A_qR2 | CGAGCAGAATGATGAAGGTG |  |
| *kir1.1* | Cyca_Kir1.1_qF2 | CCGCAGCAAAGAGGGAAAG | XM_019090270 |
|  | Cyca_Kir1.1_qR2 | CGATGGAGTGGTGATGGTGT |  |
| *atp1a1a.5* | Cyca_atp1a1a.5_qF2 | CGCCTTCCCCTACTCACTCC | LHQP01045134 |
|  | Cyca_atp1a1a.5_qR2 | GTCTCCCTCTCCACCCAACC |  |
| *rhcg1* | Cyca_Rhcg1_qF2 | CAGAAAGGAGAAGAACATAACGAG | KF051940 |
|  | Cyca_Rhcg1_qR2 | AAACCAAAGCCCACAAAGA |  |
| *ae1b1* | CycaAE1b_1_qF1 | TGGTTTGTTCCTCCGTTTGG | XM_019078606 |
|  | CycaAE1b_1_qR1 | CTCCTTGCGGTTGACGATG |  |
| *ae1b2* | CycaAE1b_2_qF1 | TCAATCGCAAGGAGCACAGA | XM_019081762 |
|  | CycaAE1b_2_qR1 | CAGCCACATACCAGGGCAGA |  |
| *atph* | Cyca_atp6v1a_qF2 | GCAGCAGAACATCTCCACAA | LHQP01024083 |
|  | Cyca_atp6v1a_qR2 | GAGCATCCATAACACCACAAGA |  |
| *ca15a* | Cyca_ca15a_qF2 | TCCACCTTCCTGTCAATCGTAAA | LHQP01036162 |
|  | Cyca_ca15a_qR2 | GCACTGTTATTTTCTCATCATCCA |  |
| *ncc* | Cyca_NCC_slc12a10.2_qF2 | CGCTCTGGTGGACTTTGTGG | XM_019086108 |
|  | Cyca_NCC_slc12a10.2_qR2 | CTGCGGGAAACTGGACTTCT |  |
| *nhe3b* | Cyca_NHE3b_qF2 | TCAGGAAGGAGTCTGTGGAG | XM_019124877 |
|  | Cyca_NHE3b_qR2 | ACCAGTCTCGCTCCCCACA |  |
| *aqp3a* | Cyca_AQP3a_qF1 | AACCTTGCTTTTGGGTTTGCT | LC069008 |
|  | Cyca_AQP3a_qR1 | CACAGGGAACTTTCTCCATTTTTC |  |
| *aqp3b* | Cyca_AQP3b_qF1 | GCTACATTGGGAATCCTTGTTTGT | LC069009 |
|  | Cyca_AQP3b_qR1 | TTTCCTCCAGGGCTCTCTCC |  |
| *ecac* | Cyca_ecac_qF2 | TTGCCCGAGGTTTTGAGATG | LHQP01039933 |
|  | Cyca_ecac_qR2 | ACCCACAAGCCAGAAGCAGA |  |
| *muc2-like* | Cyca_Muc2_like_qF2 | GCATCAACCTGCCATTCC | MF380420 |
|  | Cyca_Muc2_like_qR2 | CAGCACAGTCGTCCACCAAG |  |

**Supplementary Table 2.** Gill histology scores during the CEV infection in two strains of carp. The severity of the changes was scored with a semi‐quantitative scoring system using a scale from 0 to 3. 0 - indicates no changes, 1—mild changes, 2—moderate changes and 3 - severe changes.

| **Treatment** | **Control** |  | **Infected** |  |  |  |  |  |  |  |
| --- | --- | --- | --- | --- | --- | --- | --- | --- | --- | --- |
| **Time point** |  |  | **3 dpe** |  | **6 dpe** |  |  | **9 dpe** |  | **13 dpe** |
| **Strain / Clinical sign** | **Koi** | **AS** | **AS** | **Koi** | **AS** | **Koi** | **Koi (metabolome)** | **AS** | **Koi** | **AS** |
| **Hyperplasia of epithelial cells** | 0 (0-1) | 0 (0-1) | 0 (0-1) | 1 (0-2) | 1 (0-2) | 2 (1-3) | 1 (1-3) | 1,5 (1-2) | 2 (1-3) | 1 (1-2) |
| **Proliferation of Intralamellar cells** | 0,5 (0-1) | 0 (0-2) | 1,5 (0-2) | 1 (0-2) | 2 (0-2) | 2,5 (2-3) | 2 (2-3) | 2 (1-2) | 2 (0-3) | 2 (2-3) |
| **Apoptotic cells/ cellular debris** | 0 (0-0) | 0 (0-1) | 0 (0-1) | 0,5 (0-1) | 1 (0-1) | 2 (1-2) | 2 (1-3) | 1 (0-2) | 1 (1-2) | 1 (0-2) |
| **Infiltration of eosinophilic granular cells** | 0 (0-0) | 0 (0-1) | 0,5 (0-1) | 0,5 (0-1) | 0 (0-1) | 2 (1-2) | 3 (2-3) | 0 (0-1) | 1 (1-2) | 0 (0-1) |
| **Occlusion of intralamellar space** | 0 (0-0) | 0 (0-0) | 0 (0-0) | 0 (0-0) | 0 (0-0) | 2,5 (1-3) | 3 (3) | 0 (0-0) | 2 (0-3) | 0 (0-3) |

**Supplementary Table 3.** Gill histology scores during the CEV infection combined with the salt rescue. The severity of the changes was scored with a semi‐quantitative scoring system using a scale from 0 to 3. 0 - indicates no changes, 1—mild changes, 2—moderate changes and 3 - severe changes.

| **Treatment / Clinical sign** | **Control** | **Control salt** | **Infected Salt** | **Infected no salt** |
| --- | --- | --- | --- | --- |
| **Hyperplasia of epithelial cells** | 0 (0-1) | 0 (0-1) | 2 (2-3) | 2 (2-3) |
| **Proliferation of Intralamellar cells** | 1 (1) | 0 (0-1) | 1 (1-2) | 1 (1-2) |
| **Apoptotic cells/ cellular debris** | 0(0-1) | 0 (0-1) | 1 (1-2) | 1 (0-1) |
| **Infiltration of eosinophilic granular cells** | 0 (0) | 0 (0-1) | 0 (0-1) | 0 (0) |
| **Occlusion of intralamellar space** | 0 (0) | 0 (0) | 1 (1) | 1 (0-2) |


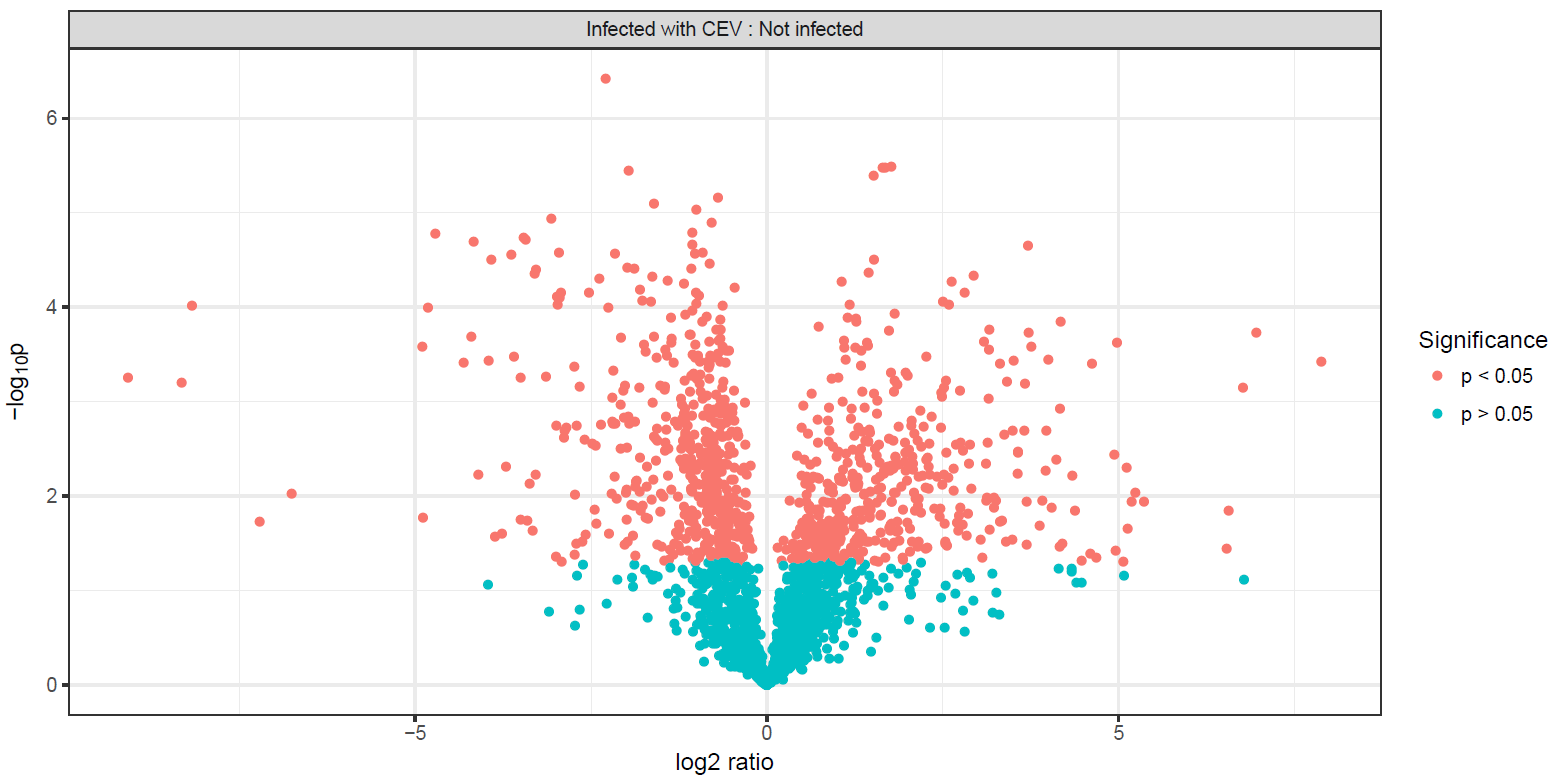


**Supplementary Fig. 1.** Volcano plot of changes in the metabolites in CEV infected large koi vs. not infected control.


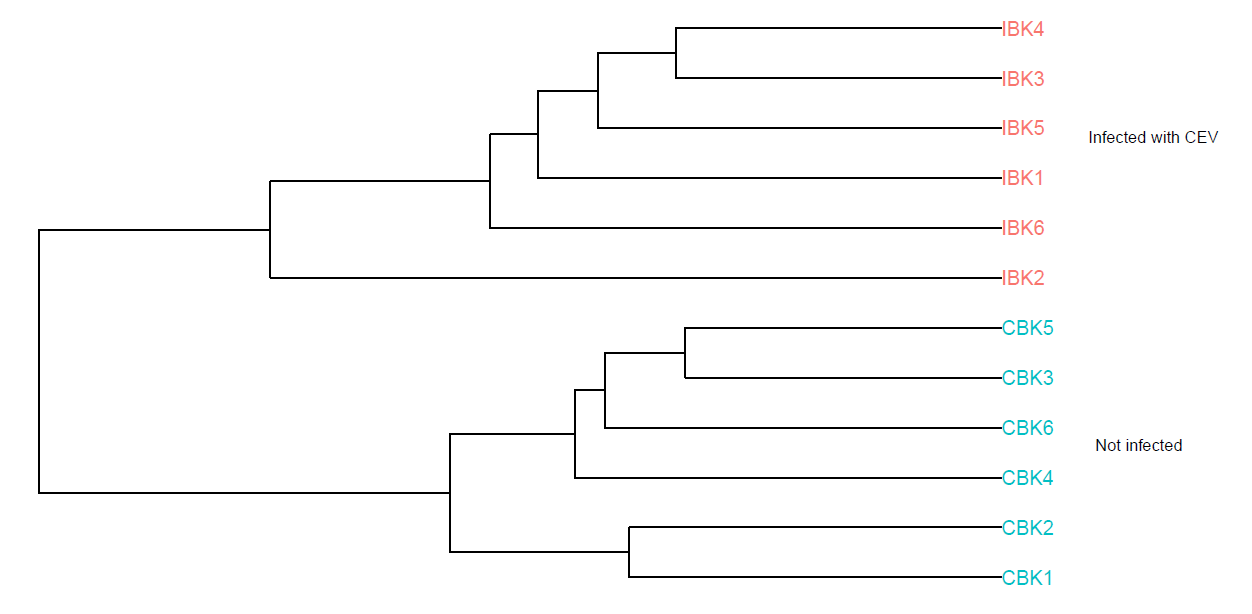


**Supplementary Fig. 2.** Dendrogram of all analysed plasma samples. IBK1-6 indicates plasma from CEV infected big koi, CBK1-6 indicated plasma from big koi before infection.


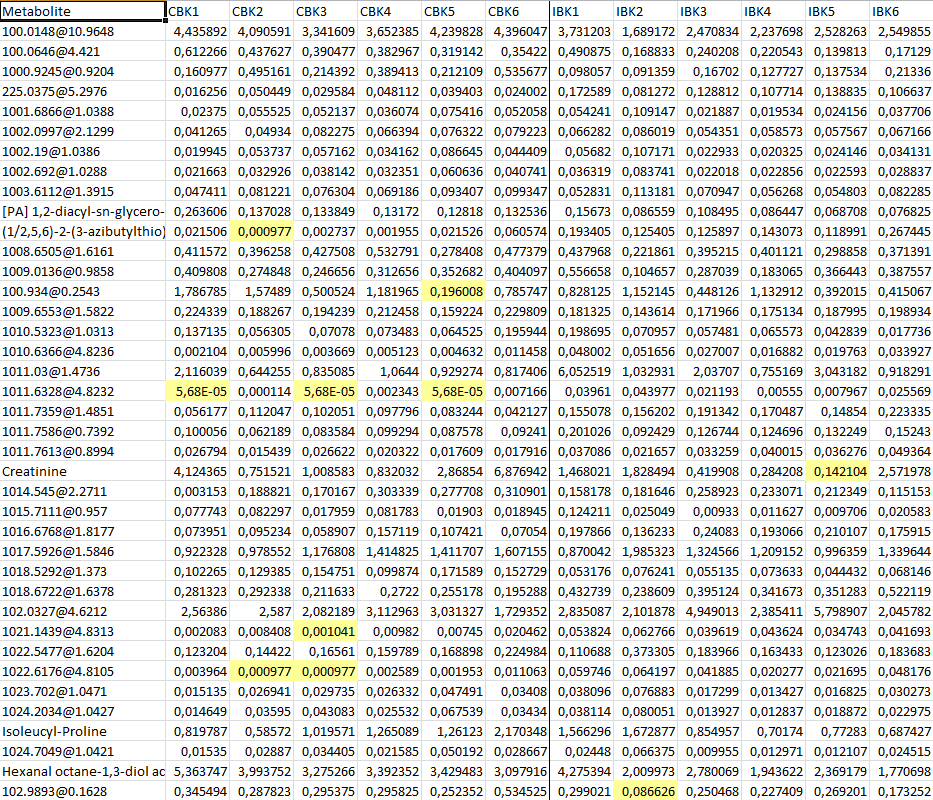


**Supplementary Fig. 3.** Table snapshot of all metabolites comparing all samples. Full version is provided in the Supplementary file 1.


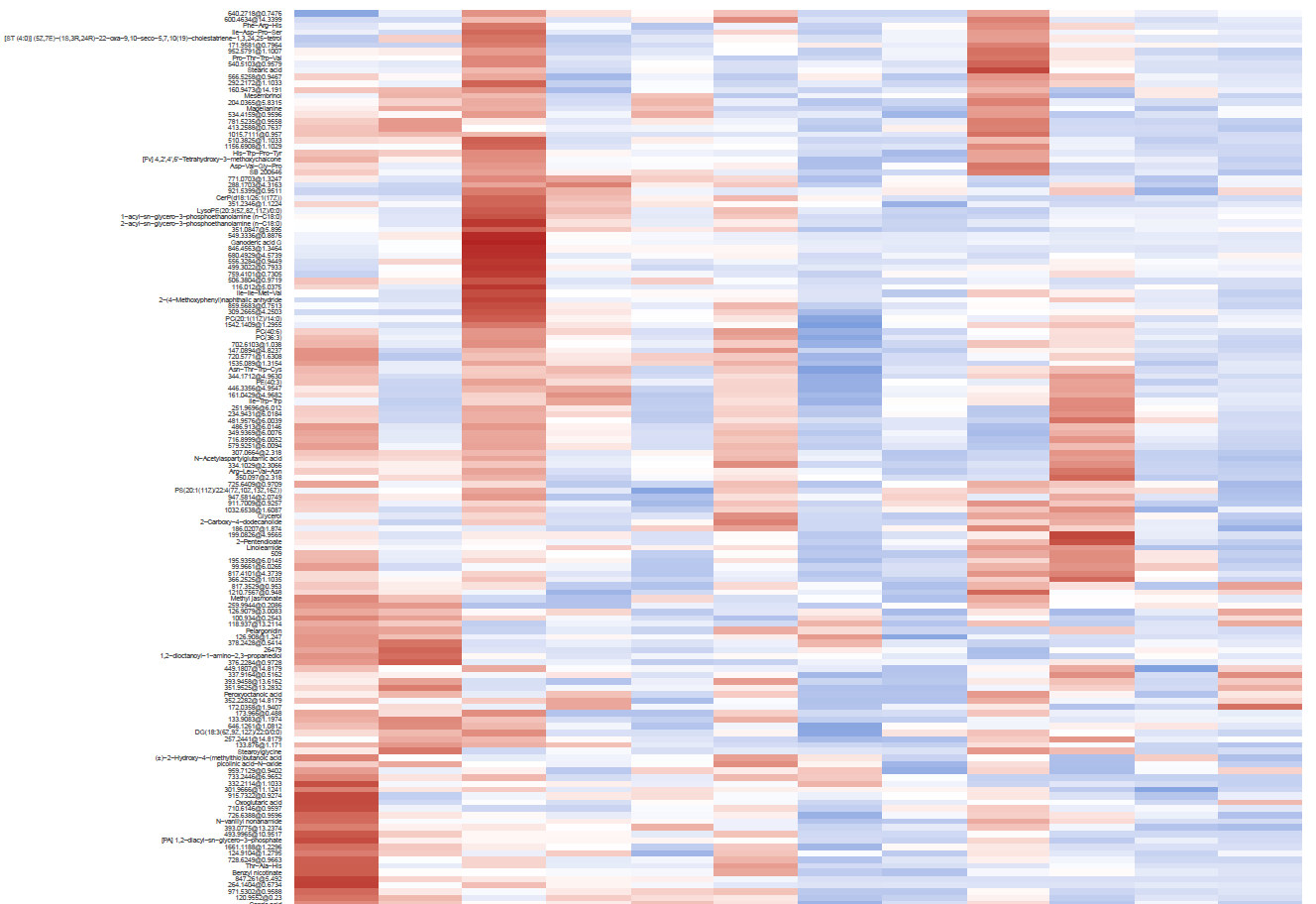


**Supplementary Fig. 4.** Heatmap snapshot of all metabolites comparing all samples. Full version is provided in the Supplementary file 2.


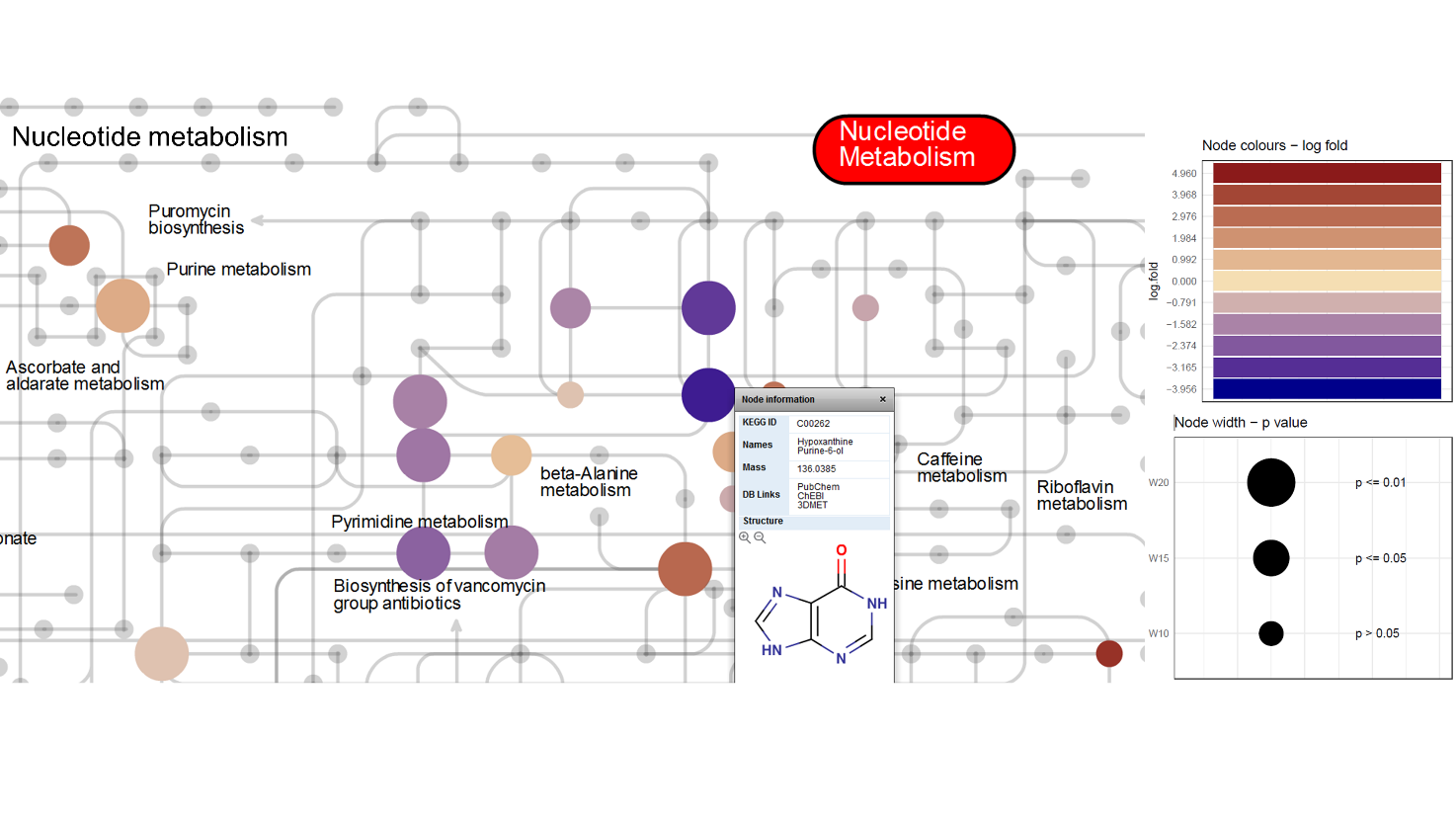


**Supplementary Fig. 5.** iPath2 snapshot of changes in nucleotide metabolism pathways. Full version is provided in the Supplementary file 3.


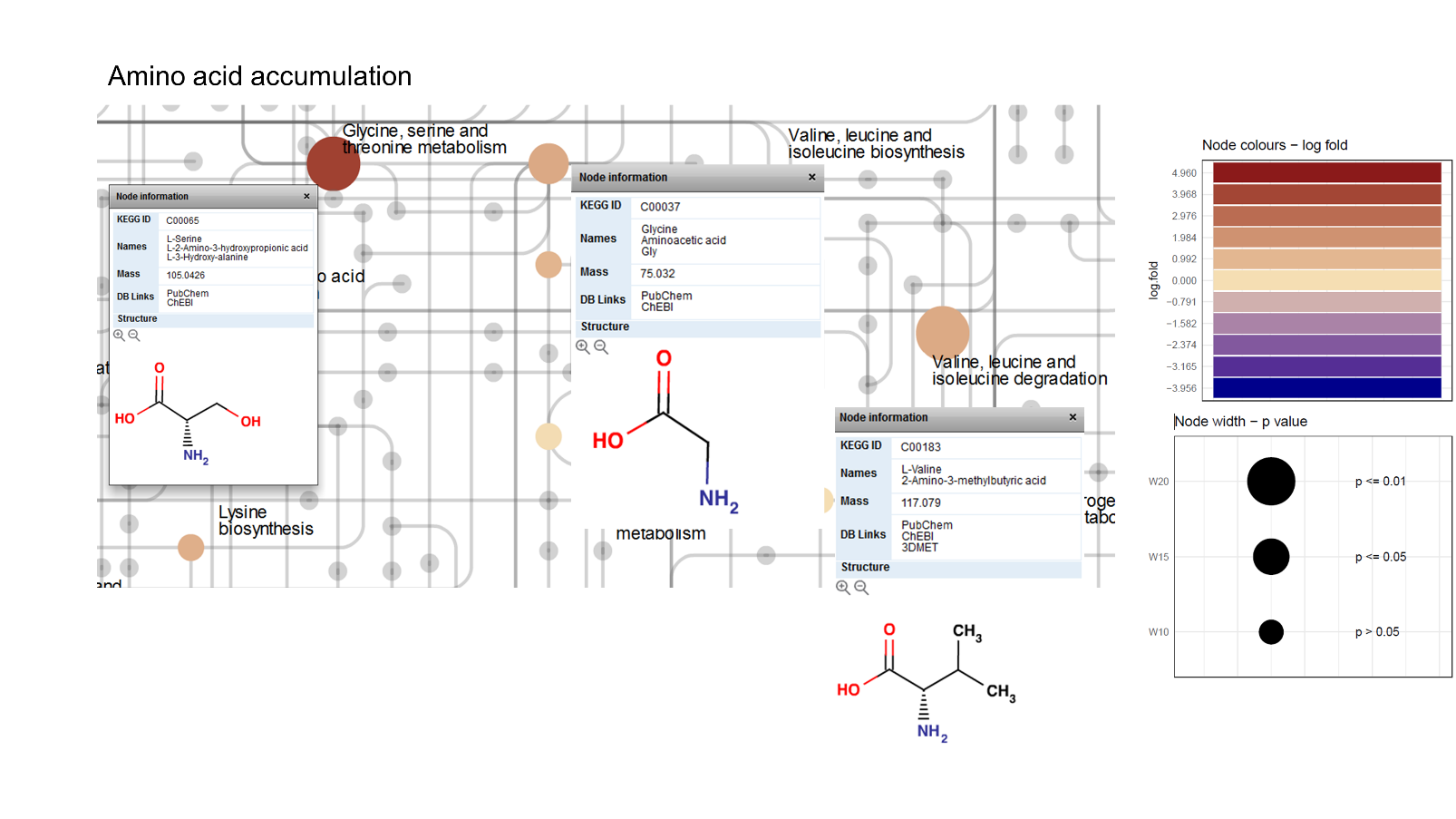


**Supplementary Fig. 6.** iPath2 snapshot of changes in amino acids metabolism pathways. Full version is provided in the Supplementary file 3.


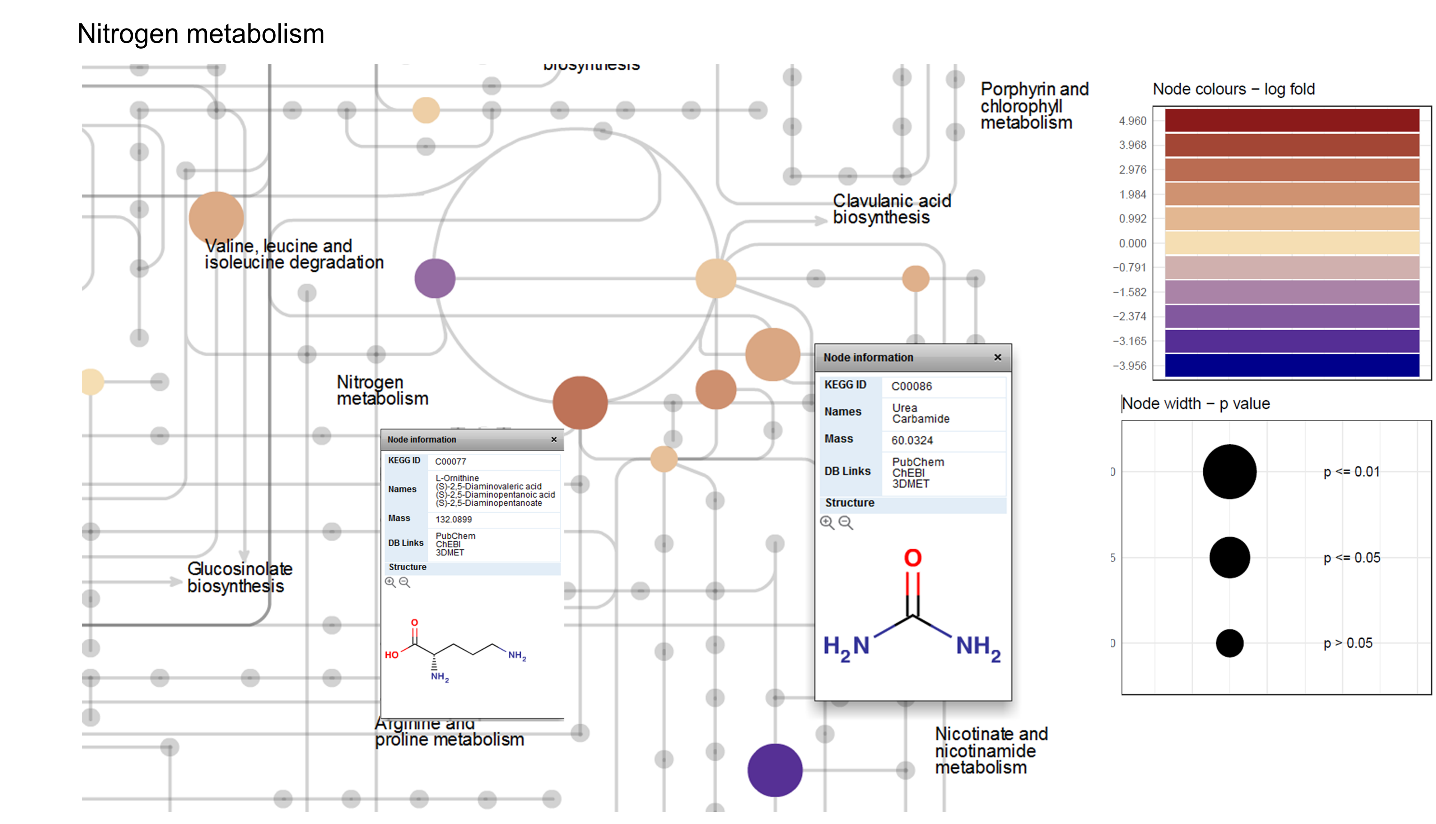


**Supplementary Fig. 7.** iPath2 snapshot of changes in nitrogen metabolism pathways. Full version is provided in the Supplementary file 3.


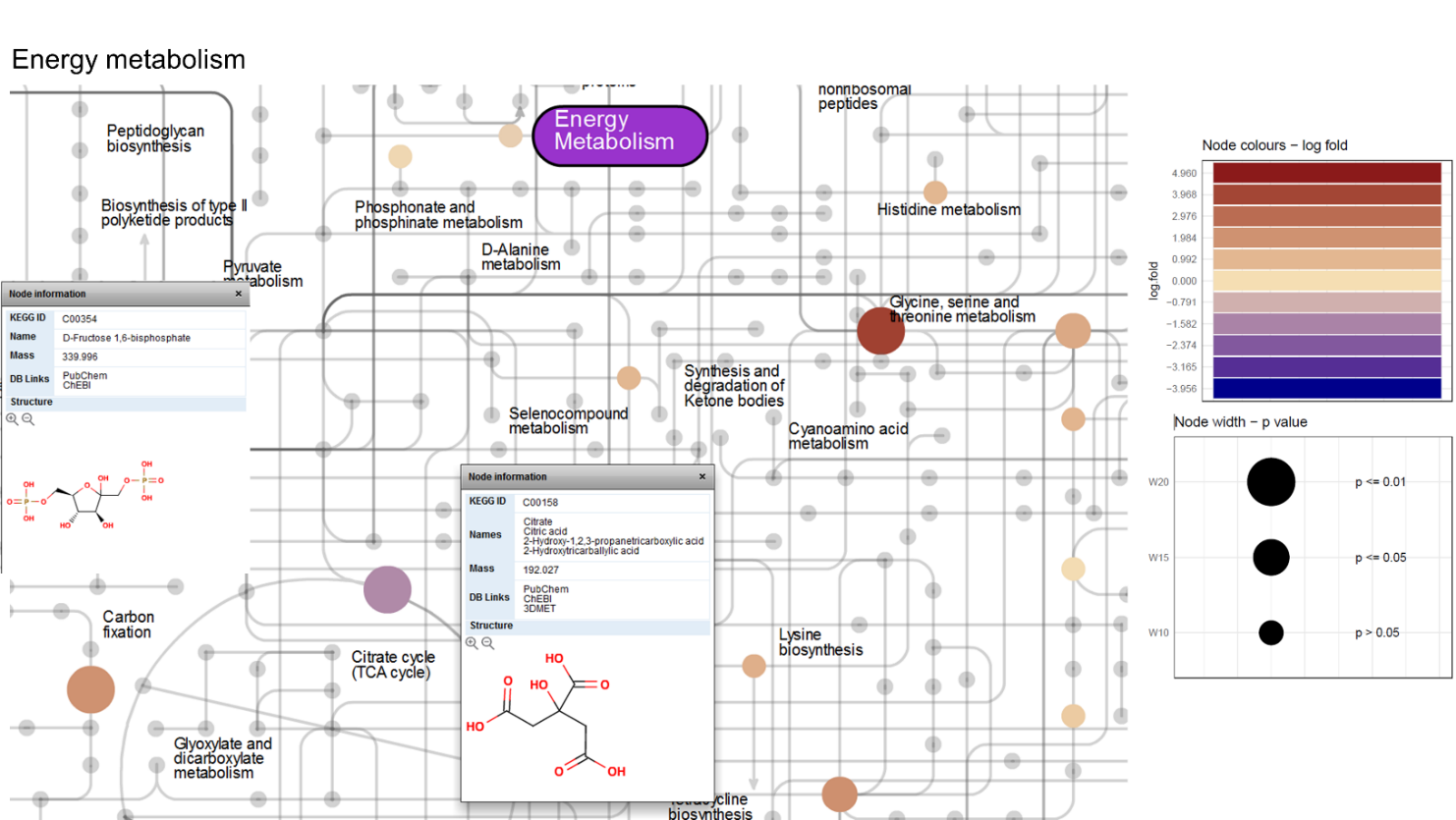


**Supplementary Fig. 8.** iPath2 snapshot of changes in energy metabolism pathways. Full version is provided in the Supplementary file 3.


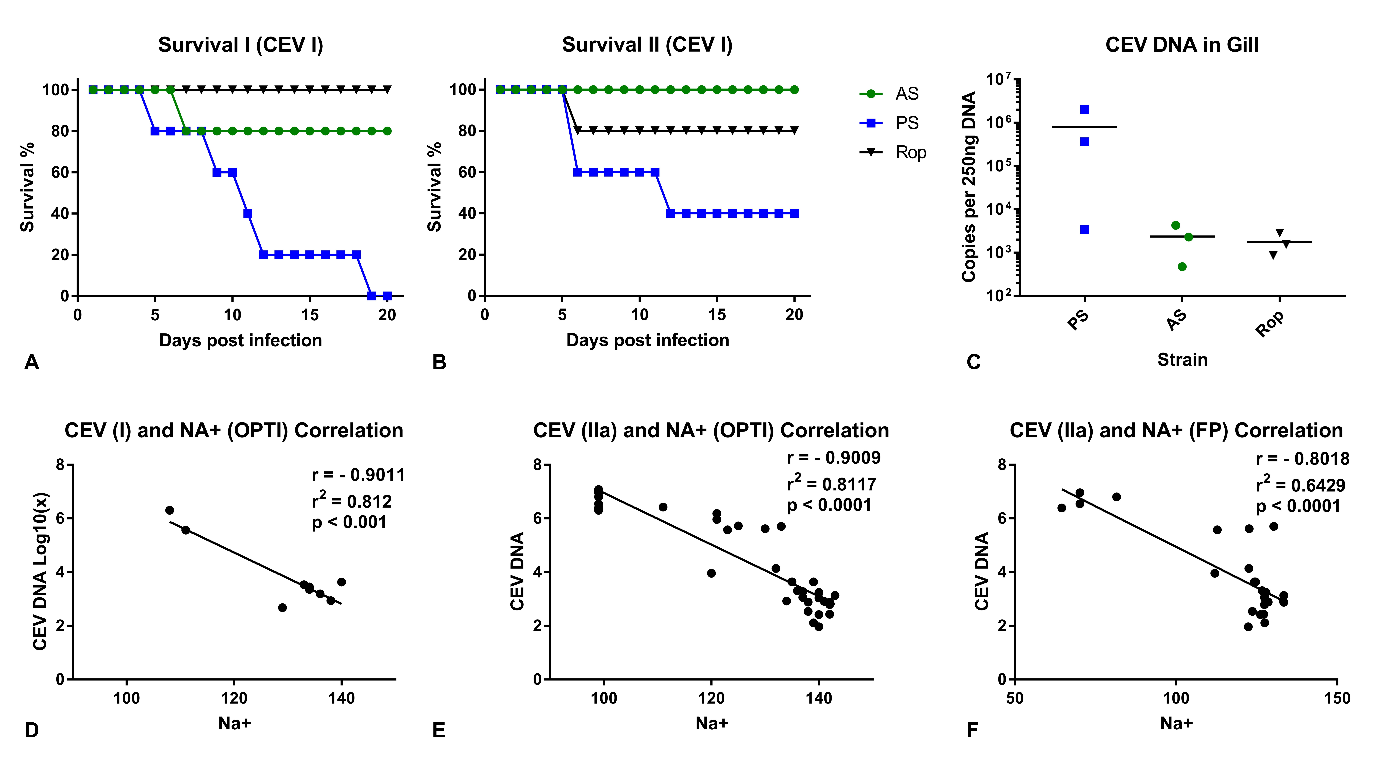


**Supplementary Fig. 9.** Kinetic of an infection of a PS, AS and Rop carp with carp edema virus from genogroup I. A and B) Mortality curves based on the number of animals removed from the experiment when they reached the clinical signs score qualifying for the humane end-point of the experiments. All animals removed presented severe sleepiness or CLB when they were not responding to external stimuli. C) CEV loads in gills of specimen from the PS, AS and Rop strains which show different susceptibility to the infection. PS were susceptible while AS and Rop were more resistant to the CEV infection and development of KSD leading to mortality. Mortality data are presented as percentage curves of fish remaining in the experiment. Virus load data are presented as single data points with an indication of mean as a horizontal line.

**Supplementary Table 4.** Blood parameters in carp infected with CEV genogroup I. Letters (a, b, c) indicate significant differences at p ≤ 0.05 between different treatments. OPTI - Osmetech OPTI CCA Blood Gas Analyzer.

| Factor | Instrument |  | PS/AS/Rop | PS | AS | Rop |
| --- | --- | --- | --- | --- | --- | --- |
|  |  |  | Control | CEV (G I) 6 dpe | CEV (G I) 6 dpe | CEV (G I) 6 dpe |
| CEV DNA | 3005p | Mean | **0a** | **7,98E+05***c** | **2,35E+03**b** | **1,75E+03**b** |
|  |  | SD | 0 | 1,08E+06 | 1,91E+03 | 1,00E+03 |
| pH | OPTI | Mean | 7,26 | 7,43 | 7,41 | 7,34 |
|  |  | SD | 0,04 | 0,07 | 0,09 | 0,04 |
| pCO_2_ (mmHg) | OPTI | Mean | 21,33 | 28,67 | 21,33 | 24,33 |
|  |  | SD | 0,58 | 4,93 | 1,53 | 2,08 |
| pO_2_ (mmHg) | OPTI | Mean | 22,00 | 39,33 | 32,67 | 32,33 |
|  |  | SD | 7,00 | 11,02 | 7,09 | 9,50 |
| BE (mmol L^-1^) | OPTI | Mean | **-15,77a** | **-4,27*c** | **-9,90b** | **-11,70b** |
|  |  | SD | 1,19 | 6,18 | 3,05 | 1,25 |
| tCO_2_ (mmol L^-1^) | OPTI | Mean | **10,07a** | **19,97*c** | **14,00b** | **13,47b** |
|  |  | SD | 0,72 | 5,78 | 1,77 | 0,71 |
| HCO_3_ (mmol L^-1^) | OPTI | Mean | **9,40a** | **19,10*c** | **13,37b** | **12,73b** |
|  |  | SD | 0,70 | 5,63 | 1,82 | 0,67 |
| Na^+^ (mmol L^-1^) | OPTI | Mean | **137,67a** | **117,33*b** | **134,33a** | **136,00a** |
|  |  | SD | 1,53 | 13,65 | 5,51 | 2,00 |
| K^+^ (mmol L-1) | OPTI | Mean | 2,27 | 2,80 | 3,00 | 3,03 |
|  |  | SD | 0,55 | 0,66 | 1,21 | 1,07 |
| Ca^++^ (mmol L^-1^) | OPTI | Mean | 1,15 | 1,15 | 1,26 | 1,32 |
|  |  | SD | 0,16 | 0,25 | 0,16 | 0,11 |
| tHb (g/L) | OPTI | Mean | 114,67 | 103,00 | 86,33 | 87,00 |
|  |  | SD | 9,02 | 3,61 | 12,01 | 7,00 |
| Hct© (%) | OPTI | Mean | 34,33 | 31,00 | 25,67 | 26,00 |
|  |  | SD | 2,52 | 1,00 | 3,51 | 2,00 |


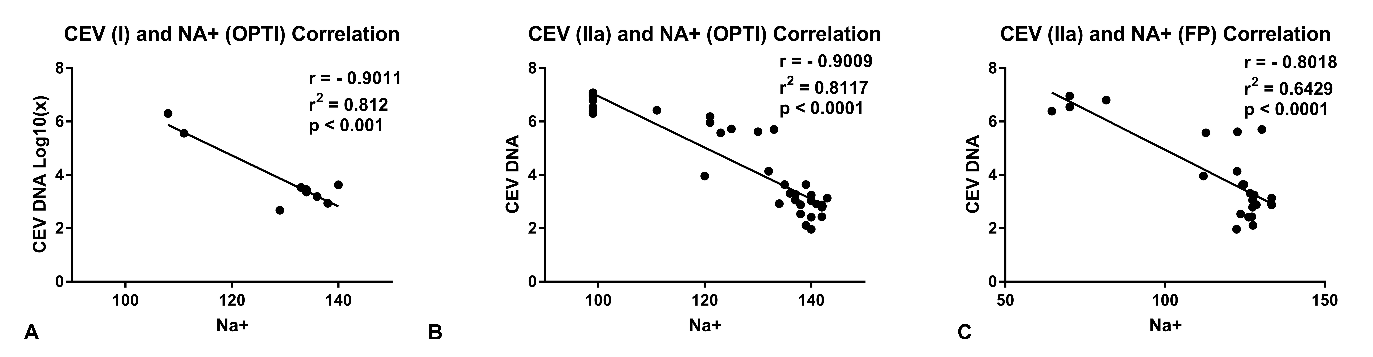


**Supplementary Fig. 10.** Pearson correlation between CEV load in gills (*p4a* DNA copies per 250 ng of extracted DNA) and sodium concentrations in blood (mmol L-1) A) results from AS, PS and Rop strains infected with CEV genogroup I (sodium measured with Osmetech OPTI CCA Blood Gas Analyzer), B) results from AS, and koi strains infected with CEV genogroup IIa (sodium measured with Osmetech OPTI CCA Blood Gas Analyzer), C) results from AS, and koi strains infected with CEV genogroup IIa (sodium measured performed by flame photometer.
