# Supplementary figures and images for: Koi sleepy disease as a pathophysiological and immunological consequence of a branchial infection of common carp with carp edema virus
Source: Virulence. 2021 Jul 16;12(1):1855–83. doi: 10.1080/21505594.2021.1948286 (PMC8288041; doi:10.1080/21505594.2021.1948286)

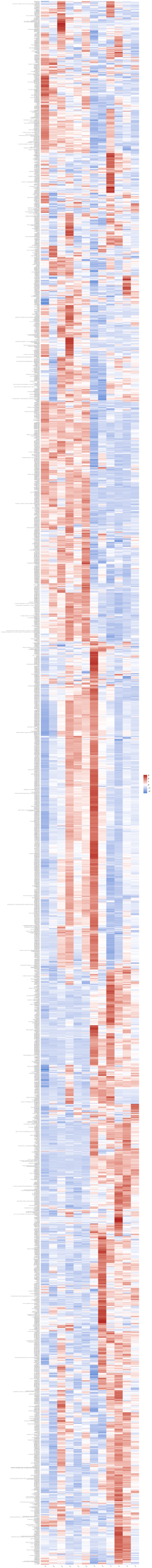

Supplement: Supplemental Material [file KVIR_A_1948286_SM6432.zip › supplementary/downloadFromZipFile.pdf]
